# Glucose starvation induces LKB1-AMPK-mediated MMP-9 expression in cancer cells

**DOI:** 10.1038/s41598-018-28074-w

**Published:** 2018-07-04

**Authors:** Hitoshi Endo, Satoshi Owada, Yutaka Inagaki, Yukari Shida, Masayuki Tatemichi

**Affiliations:** 1 Center for Molecular Prevention and Environmental Medicine, Department of Preventive Medicine, Tokai University School of Medicine, 143 Shimokasuya, Isehara, Kanagawa 259-1193 Japan; 2 Center for Matrix Biology and Medicine, Department of Regenerative Medicine, Tokai University School of Medicine, 143 Shimokasuya, Isehara, Kanagawa 259-1193 Japan

## Abstract

Cancer cells utilise the glycolytic pathway to support their rapid growth and proliferation. Since cells in most solid tumours are subjected to severe microenvironmental stresses including low nutrient and oxygen availability, such cancer cells must develop mechanisms to overcome these unfavourable growth conditions by metabolic adaptation. Although the liver kinase B1 (LKB1)-adenosine monophosphate-activated kinase (AMPK) signalling pathway plays a pivotal role in maintaining energy homeostasis under conditions of metabolic stress, the role of LKB1-AMPK signalling in aiding cancer cell survival and in malignant tumours has not yet been fully elucidated. We show that glucose starvation promotes cancer cell invasiveness and migration through LKB1-AMPK-regulated MMP-9 expression. Most intriguingly, triggering the LKB1-AMPK signalling pathway by glucose starvation-induced oxidative stress facilitates selective autophagy, which in turn enhances Keap1 degradation and the subsequent activation of Nrf2. Following this, Nrf2 regulates the transactivation of MMP-9 via Nrf2 binding sites in the promoter region of the MMP-9 gene. These mechanisms also contribute to the suppression of excessive oxidative stress under glucose starvation, and protect against cell death. Our data clearly shows that LKB1-AMPK signalling not only maintains energy and oxidative stress homeostasis, but could also promote cancer progression during metabolic stress conditions by MMP-9 induction.

## Introduction

Cancer cells exhibit significant alterations in metabolic pathways that support cell mass accumulation, nucleic acid biosynthesis, and mitotic cell division^1,2^. Unlike normal cells, cancer cells preferentially utilise the glycolytic pathway even in the presence of oxygen^3^. Sufficient glucose supply facilitates rapid tumour growth through the generation of intermediates that are required for the synthesis of essential cellular components^4^. However, as most solid tumours tend to outgrow existing vasculature, cells in such tumours experience stressful microenvironments characterised by low nutrient and oxygen levels. For example, glucose concentrations in human colon and gastric cancer tissues have been shown to be significantly lower than those in surrounding non-cancerous tissues^5^. Therefore, in order to survive in such unfavourable microenvironments, cancer cells must adapt and escape to sites with more favourable growth conditions. In addition, several studies have shown that cancer cells which survive such gruelling stresses form tumours with highly malignant phenotypes^6,7^.

The liver kinase B1 (LKB1)-adenosine monophosphate-activated kinase (AMPK) signalling pathway is a key energy sensor in normal and cancer cells that plays a central role in sensing energy availability in the cell; it also induces metabolic adaptation pathways to ensure cell survival. During nutrient deprivation and hypoxia, which lead to energetic stress conditions that are sensed through elevated ratios of intracellular AMP/ATP, AMP-activated protein kinase (AMPK), a serine/threonine protein kinase, is activated by liver kinase B1 (LKB1) via phosphorylation^8,9^. Once activated, the LKB1-AMPK signalling pathway increases catabolic ATP-generating processes, such as glycolysis and fatty-acid oxidation, and inhibits ATP-consuming biosynthetic processes such as protein, cholesterol, and fatty acid synthesis^10,11^. Although hyper-activation of the LKB1-AMPK signalling pathway is associated with anti-tumourigenic effects^11^, several studies have now indicated that physiological LKB1-AMPK activation contributes to pro-tumourigenic effects^12–15^. However, how LKB1-AMPK-mediated adaptation to stressful microenvironments can cause cancer cells to develop malignant phenotypes has not yet been elucidated.

The aggressive growth and metastatic spread of cancer cells is a hallmark of malignant tumours, and results in high mortality among cancer patients^16^. For tumour progression through invasion and metastasis, cancer cells within tumours must adapt to stressful microenvironments that are characterised by oxygen or nutrient deficiencies, local acidosis, and the presence of elevated levels of reactive oxygen species (ROS)^17,18^. Since excessive levels of ROS can cause cell death, cancer cells must regulate ROS levels to maintain the intracellular redox balance in order to survive in the ROS-rich tumour microenvironment^17,19^. Recent work has indicated that the metabolic sensor, AMPK, can also be activated by ROS through upstream signalling kinases, including LKB1, and could help in preventing ROS-induced apoptosis^20,21^. The LKB1-AMPK pathway promotes cell survival during glucose starvation by either inhibiting the mammalian target of rapamycin (mTOR) or by activating the tumour suppressor p53^22,23^. Besides this, AMPK also promotes cancer cell survival by regulating intracellular NADPH homeostasis during metabolic stress caused by glucose starvation^24^. Accumulating evidence further suggests that AMPK activation could be important for the development of malignant tumour characteristics in several types of cancer^12–15^. However, it remains to be determined if the protective effects of the LKB1-AMPK signalling pathway under oxidative stress and glucose starvation conditions can affect cancer cell migration and invasiveness.

Cancer progression involves multiple processes that include the loss of adhesion between cells and extracellular matrix (ECM), proteolytic degradation of the ECM, extravasation leading to invasion into new tissues, and finally, colonisation^16,25^. Matrix metalloproteinases (MMPs) secreted by tumour cells, stromal fibroblasts, or infiltrating inflammatory cells, have been strongly implicated in multiple stages of the invasive and metastatic progression of tumour cells as they are involved in the degradation of the vascular basement membrane and remodelling of the ECM during angiogenesis^25^. In particular, matrix metalloproteinase-9 (MMP-9) is not only important for invasion and metastasis, but is also involved in tumour angiogenesis^26^. Therefore, it is reasonable to hypothesise that expression of MMP-9 could help cancer cells to escape the microenvironmental stresses of nutrient deprivation and that expression of MMP-9 may promote metastatic cancer progression.

Although cancer progression is considered to be a part of adaptation to microenvironmental stresses, the role played by the LKB1-AMPK signalling pathway in the development of malignant characteristics in cancer cells, especially those involving invasiveness and migration under nutrient stress conditions, remain poorly understood. This lacuna in our knowledge prompted us to investigate the molecular mechanisms that could lead to cancer progression during glucose starvation, especially those connected to the activation of LKB1-AMPK-mediated MMP-9 induction. Since most malignant tumours have the common characteristics of high invasiveness, metastatic potential, and adaptability to circumstances, even if they arise from highly diverse genetic backgrounds and development sites^27^, this study is likely to contribute significantly to our understanding of the mechanisms of adaptation to microenvironmental stresses that may be common in and highly specific to aggressive cancers.

## Results

### LKB1-AMPK pathway is involved in cellular migration, invasion, and inducing MMP-9 expression in response to glucose starvation

**Figure 1:**
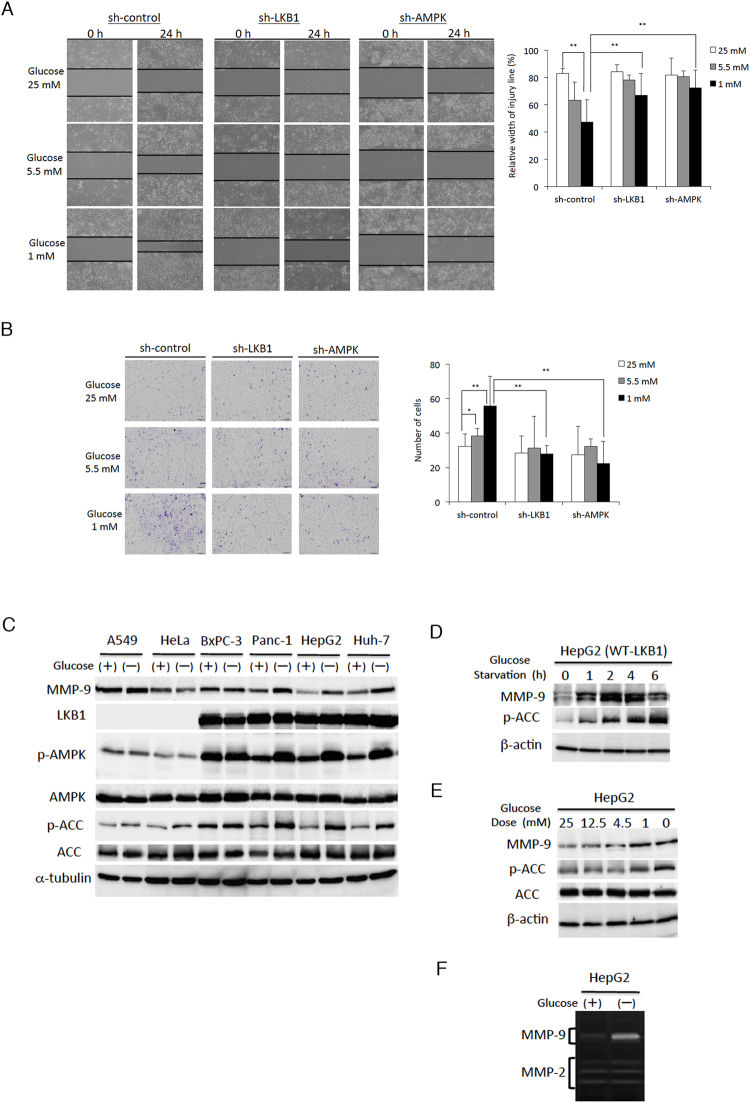
The LKB1-AMPK signalling pathway enhances cancer cell migration and invasiveness, and induces MMP-9 expression in response to glucose starvation. (**A**) HepG2 cells grown to more than 90% confluence were treated with mitomycin C for 3 h and then wounded by scraping. Cells stably expressing control-shRNA (sh-control), LKB1-shRNA (sh-LKB1), or AMPK-shRNA (sh-AMPK) were incubated in the presence of 25, 5.5, and 1 mM glucose, and observations on cell migration were made using phase-contrast microscopy after 0 and 24 h of incubation in the indicated glucose concentrations. Based on the width of the wound at 0 h, the relative width at 24 h was calculated and expressed as the mean ± SD of four selected fields. Data are expressed as mean ± SD of 3 independent experiments. (**B**) Cells were incubated in the presence of 25, 5.5, and 1 mM glucose for 24 h, following which invasiveness was measured using matrigel transwell assays. Cells that had migrated to the lower surface of the transwell membrane were stained with haematoxylin. Data are representative of three independent experiments, and are expressed as the mean ± SD of cell numbers from 5 random fields per filter in duplicate. (**C**) Western blots of protein extracts obtained from various cancer cell lines incubated in glucose-free medium in the absence (−) or presence (+) of 5.5 mM glucose for 6 h. (**D**) Western blots of protein extracts obtained from HepG2 cells starved of glucose for the indicated time periods. (**E**) Western blots of protein extracts obtained from HepG2 cells incubated in the indicated concentrations of glucose. Western blot analyses were performed with specific antibodies against the indicated proteins, with either α-tubulin or β-actin as loading controls. (**F**) HepG2 cells were incubated in glucose-free medium in the absence (−) or presence (+) of 5.5 mM glucose for 24 h. Conditioned culture media were analysed for gelatinolytic activities of MMP-9 and MMP-2 by zymography. Uncropped blots of all experiments are presented in Supplementary Fig. 9. *P < 0.05; **P < 0.01.

Starvation-based cancer microenvironmental stresses are important drivers for the development of malignant characteristics in tumours^6,7^. As the LKB1-AMPK signalling pathway is known to contribute to the survival of cancer cells under conditions of nutrient deprivation^9^, it is possible that this pathway also plays a role in accelerating cancer cell progression during energy stress. However, this aspect of the LKB1-AMPK signalling pathway’s role in cancer progression has not been explained. To investigate the role of LKB1-AMPK signalling in the development of malignancy, we first examined LKB1-AMPK-mediated cell migration and invasiveness under glucose starvation conditions. Cell migration and invasiveness were dramatically enhanced in the cancer cell line HepG2 with decreasing glucose concentrations; migration and invasiveness were inhibited if LKB1 or AMPK were silenced (Fig. 1A,B). However, the proliferative capacity of cells expressing LKB1 or AMPK shRNA was not lowered, and instead showed a significant increase (Fig. S1). It is unsurprising to see that silencing LKB1 or AMPK in cells does not inhibit their proliferative capacity, since the LKB1-AMPK pathway is also known to act as an inhibitor cell proliferation through the stabilisation of p53^23^. Our results indicate that cancer progression under the microenvironmental stress induced by low nutrient conditions is dependent on LKB1-AMPK activation.

MMP-9 is known to play an essential role in enhancing the migration and invasive capacities of malignant tumour cells by degrading the surrounding ECM, which helps to accelerate the process of metastasis^25^. In order to investigate if MMP-9 is involved in enhancing the migration and invasive capacity of cancer cells induced by glucose starvation, we examined the expression of MMP-9 in glucose-starved cells, and tried to determine if this expression of MMP-9 was dependent on the activation of the LKB1-AMPK signalling pathway in several human cancer cell lines. The expression of MMP-9 and the phosphorylation of AMPK and acetyl-CoA carboxylase (p-ACC) (a direct AMPK substrate) were found to be induced by glucose starvation in the pancreatic ductal carcinoma cell line Panc-1, and the hepatoma cell lines HepG2 and Huh-7 (Fig. 1C). Although MMP-9 expression and AMPK activation were constitutively observed in the other pancreatic ductal carcinoma cell line BxPC-3, extended exposure time of glucose starvation also caused prominent induction of MMP-9 expression and AMPK phosphorylation (Fig. S2A). However, these results were not obtained in the LKB1-deficient A549 (a non-small cell lung carcinoma cell line) and HeLa (a cervical adenocarcinoma cell line) cells. AMPK activation and MMP-9 expression were correlated and induced by glucose starvation in a time-dependent and glucose concentration-dependent manner in HepG2 cells (Fig. 1D,E), but not in HeLa cells (Fig. S2B,C). Furthermore, gelatin zymography indicated that the gelatinolytic activity of MMP-9, but not MMP-2, was markedly increased by glucose starvation (Fig. 1F). These results suggest that the LKB1-AMPK signal plays an important role in the expression of MMP-9 under glucose starvation.

### ROS contribute to LKB1-AMPK-mediated induction of MMP-9 expression, but do not affect cellular energy levels during glucose starvation

**Figure 2:**
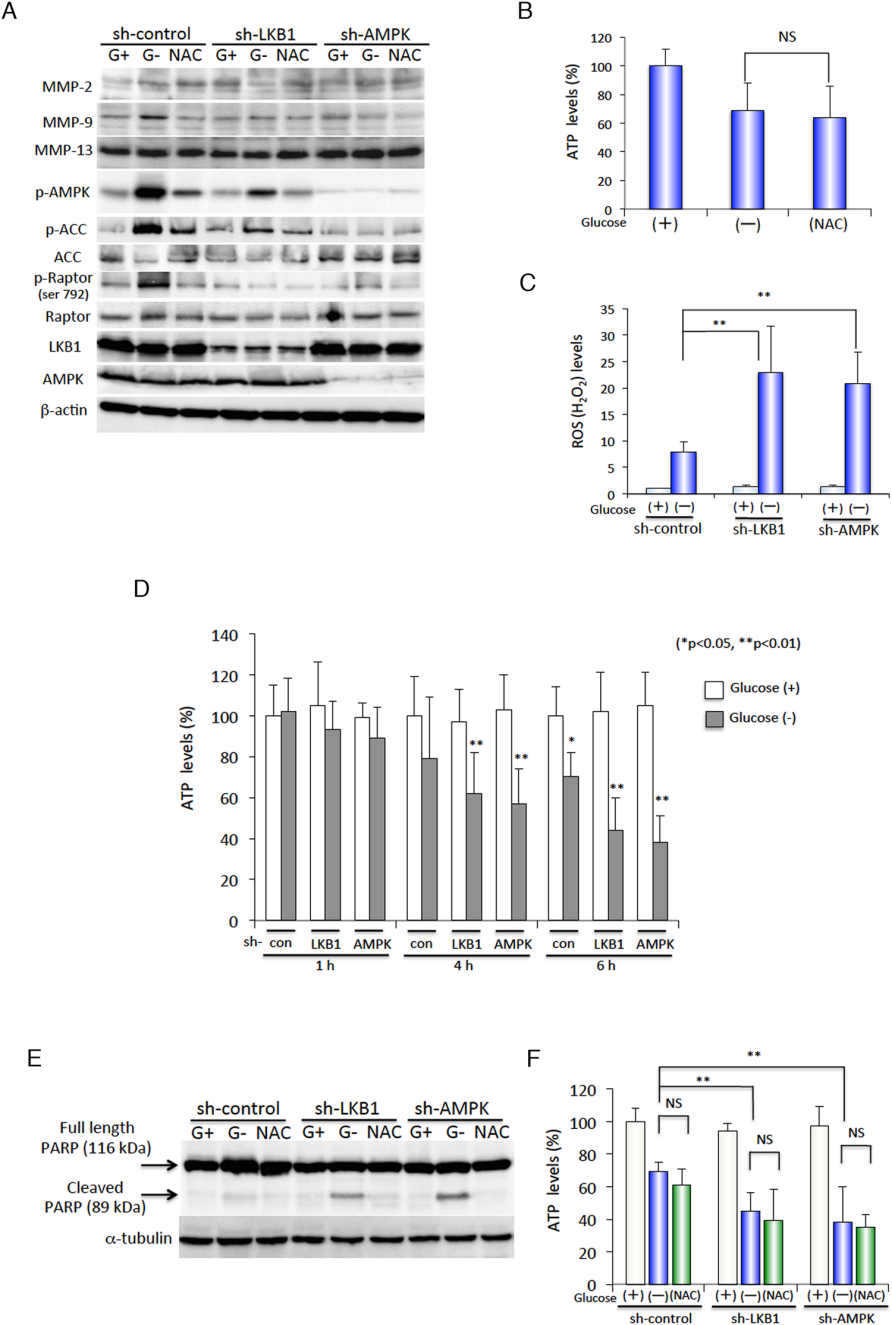
ROS-triggered LKB1-AMPK-mediated MMP-9 expression during glucose starvation is independent of decreases in ATP levels. (**A**) HepG2 cells expressing sh-control, sh-LKB1, or sh-AMPK were incubated in glucose-free medium in the absence (G−) or presence (G+) of 5.5 mM glucose, or 2.5 mM NAC for 6 h. Western blot analysis was performed on protein extracts of these cells with antibodies against the indicated proteins with β-actin as a loading control. (**B**) Intracellular ATP levels were measured in HepG2 cells after incubation in glucose-free medium in the absence (−) or presence (+) of 5.5 mM glucose, or 2.5 mM NAC for 6 h. Data are expressed as percentage changes in mean ATP values from those obtained for cells grown in the presence of 5.5 mM glucose. (**C**) ROS (H_2_O_2_) levels measured in HepG2 cells expressing sh-control, sh-LKB1, or sh-AMPK and incubated in glucose-free medium in the absence (−) or presence (+) of 5.5 mM glucose for 6 h. Data are normalised for cell numbers and expressed as percentage changes in mean ROS values from those obtained for cells expressing sh-control and grown in the presence of 5.5 mM glucose (+). (**D**) Intracellular ATP levels were measured in HepG2 cells expressing sh-control, sh-LKB1, or sh-AMPK incubated in glucose-free medium in the absence (−) or presence (+) of 5.5 mM glucose for the indicated time periods. (**E**) Whole-cell extracts from HepG2 cells expressing sh-control, sh-LKB1, or sh-AMPK after incubation in glucose-free medium in the absence (−) or presence (+) of 5.5 mM glucose, or 2.5 mM NAC for 8 h were subjected to western blot analysis using anti-PARP and anti-α-tubulin antibodies. (**F**) Intracellular ATP levels were measured in HepG2 cells expressing sh-control, sh-LKB1, or sh-AMPK and incubated in glucose-free medium in the absence (−) or presence (+) of 5.5 mM glucose, or 2.5 mM NAC for 6 h. The data are presented as mean ± SD of 3 independent sets of experiments. Uncropped blots for all experiments are presented in Supplementary Fig. 9. For (**B**–**D**), and (**F**), all data are expressed as mean ± SD of 3 independent experiments. NS, not significant; *P < 0.05; **P < 0.01.

Glucose limitation could cause oxidative stress by decreasing NADPH generation in the pentose phosphate pathway^28^. Furthermore, ROS produced by low-nutrient metabolic stress are well-known to induce AMPK activation^20,21^, and LKB1 is known to play an important role in protecting the cell against ROS-mediated damage^29^. Based on these connections between LKB1, AMPK, and ROS, we hypothesised that in glucose-starved conditions, certain levels of ROS may stimulate the induction of MMP-9 expression via LKB1 and/or AMPK activation. To test this possibility, under glucose starvation conditions, we treated HepG2 cells with NAC, an ROS scavenger that inhibits oxidative stress (Fig. 2A). NAC treatment completely abolished MMP-9 induction by glucose starvation in control vector-transfected cells, whereas MMP-2 and MMP-13 expression levels remained unchanged. Treatment with other antioxidants such as Trolox (a water-soluble analog of vitamin E) and ascorbic acid (vitamin C) also substantially reduced glucose starvation-induced MMP-9 expression (Fig. S3A,B). We also found that NAC treatment inhibited the glucose starvation-induced phosphorylation of AMPK and its downstream targets, ACC and Raptor. Furthermore, glucose starvation-induced MMP-9 expression was severely diminished by LKB1 or AMPK shRNA expression. As activation of the LKB1-AMPK signalling pathway is known to maintain intracellular ATP levels under conditions of energy stress^8^, we also conducted experiments to determine if the reductions in intracellular ATP levels could be rescued by NAC treatment. Unexpectedly, treatment with NAC did not restore ATP levels to normal from the significantly reduced ATP levels during glucose starvation (Fig. 2B). Based on these observations, we conclude that under glucose starvation conditions, oxidative stress plays an important role in the initial activation of the LKB1-AMPK signalling pathway; the decrease in ATP levels under these conditions, however, does not seem to play any part in LKB1-AMPK activation.

Following this, we then investigated the role of LKB1 and AMPK on the regulation of oxidative stress, for which, we examined the levels of the major ROS, hydrogen peroxide (H_2_O_2_), in cells under glucose starvation conditions (Fig. 2C). Interestingly, glucose starvation induced a substantial degree of ROS generation; LKB1 or AMPK knockdown cells exhibited significantly higher ROS levels than control cells under glucose starvation. In addition, knockdown of LKB1 or AMPK significantly reduced intracellular ATP levels under glucose starvation in a time-dependent manner (Fig. 2D). No significant differences in ATP levels were observed in LKB1 or AMPK knockdowns at normal glucose concentrations (Fig. 2D). Consistent with these results, the suppression of LKB1 or AMPK in cells made them more sensitive to glucose starvation-induced apoptosis with a marked increase in the cleavage of the apoptotic marker poly (ADP-ribose) polymerase-1 (PARP-1) (Fig. 2E). Quite unexpectedly, treatment with NAC did not further increase levels of cleaved PARP-1 in sh-control cells (Fig. 2E) under glucose starvation conditions despite suppressed AMPK phosphorylation (Fig. 2A). Furthermore, glucose starvation-induced PARP-1 cleavage was completely inhibited by NAC treatment even if LKB1 and AMPK were silenced. However, treatment with NAC also did not restore ATP levels in LKB1 or AMPK knockdowns to normal from the significantly reduced ATP levels during glucose starvation (Fig. 2F). Therefore, it is likely that glucose starvation-mediated cell death is caused by excessive, uncontrolled ROS generation, but is of little relevance to the ATP reduction in LKB1 or AMPK knockdown cells. Taken together, these results clearly indicate that LKB1-AMPK activation is not only essential for the maintenance of ATP levels, but also plays a prominent role in controlling ROS levels; furthermore, these ROS levels, in turn, are important for cell survival and induction of MMP-9 expression under glucose starvation conditions.

### Transcriptional activation of MMP-9 is regulated by Nrf2 under glucose starvation

**Figure 3:**
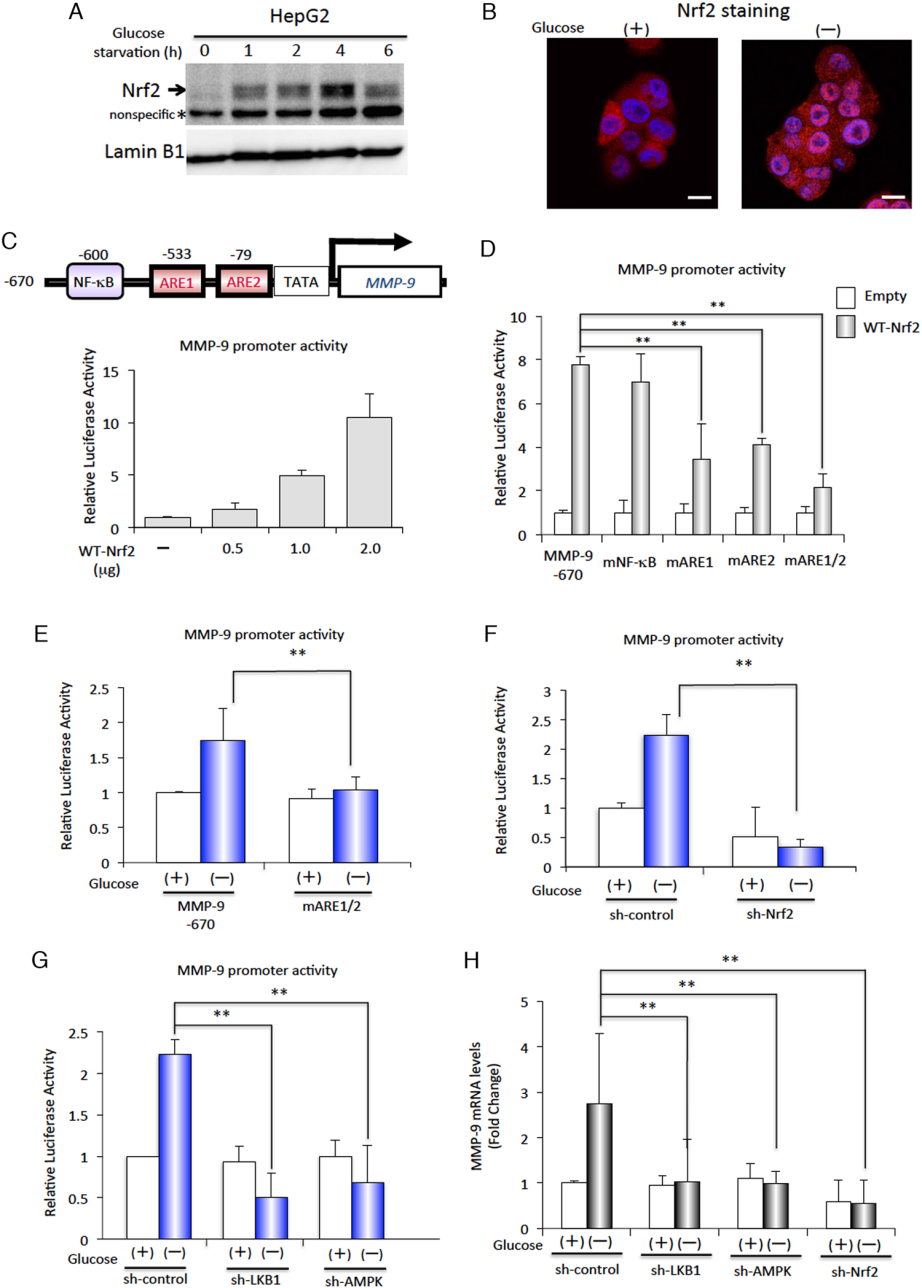
Glucose starvation-induced Nrf2 regulates transcription of the MMP-9 gene. (**A**) HepG2 cells were incubated in glucose-free medium for the indicated time periods, and the expression of Nrf2 was detected by western blot analysis; anti-Lamin B1 was used as a marker for nuclear extracts. The asterisk indicates a nonspecific band. Uncropped blots for this experiment are presented in Supplementary Fig. 9. (**B**) Cellular localisation of Nrf2 was determined by immunofluorescence staining after treatment with (+) or without (−) glucose for 4 h. DAPI staining was performed to identify the nucleus. The scale bar represents 10 μm. (**C**) A schematic of the MMP-9 promoter region is shown in the upper panel. Cells were transfected with either empty luciferase vector or different concentrations of the WT-Nrf2 expression vector, together with the MMP-9 promoter-luciferase vector. The luciferase activity in cells transfected with the empty luciferase vector was used to determine basal levels of luciferase activity, and set as 1 for this experiment and for future luciferase assays as well. (**D**) Luciferase activities of the luciferase reporter constructs containing wild type (−670) MMP-9 promoter and MMP-9 promoters containing site-specific mutants of the NF-κB binding site (mNF-κB), ARE1 (mARE1), ARE2 (mARE2), and multiple-site mutations of ARE1 and ARE2 (mARE1/2) (**E**) Luciferase activities of wild type (−670) MMP-9 promoter and mutants of ARE1 and ARE2 regions (mARE1/2) were measured after treatment with (+) or without (−) glucose for 6 h. (**F**) HepG2 cells were transfected either with sh-control or sh-Nrf2, together with the MMP-9 promoter-luciferase construct. After transfection, cells were incubated in the absence (−) or presence (+) of 5.5 mM glucose for 6 h. (**G**) HepG2 cells were transfected with sh-control, sh-LKB1, or sh-AMPK, together with the MMP-9 promoter- luciferase construct. After transfection, cells were incubated in the absence (−) or presence (+) of 5.5 mM glucose for 6 h. (**H**) HepG2 cells were transfected with sh-control, sh-LKB1, sh-AMPK, or sh-Nrf2. After transfection, cells were incubated in the absence (−) or presence (+) of 5.5 mM glucose for 6 h. *MMP-9* mRNA expression levels were examined by real-time RT-PCR. Relative expression was determined using β-actin as an internal control gene. For all experiments, the data are presented as mean ± SD of 3 independent experiments. **P < 0.01.

Nuclear factor E2-related factor 2 (Nrf2) also known as nuclear factor (erythroid-derived 2)-like 2 (NFE2L2) is a transcription factor that plays a critical role in activating antioxidant defences in response to oxidative stresses; Nrf2 does this by inducing the expression of numerous cytoprotective genes through the antioxidant response element (ARE; TGA(G/C)NNNGC) in the *cis*-acting element of the target genes’ promoters^30,31^. Consistent with an earlier report^32^, our western blotting and immunostaining analyses showed that a rapid increase in the expression of Nrf2 within the nucleus is induced by glucose starvation (Fig. 3A,B). To investigate the relationship between Nrf2 expression and MMP-9 induction by glucose starvation, we examined the reported promoter sequence of human MMP-9^33^. Interestingly, the proximal region of the MMP-9 gene promoter contains two putative ARE sequences and an NF-κB binding site (Fig. 3C, upper panel). To confirm that these AREs function as Nrf2-responsive transcription control elements, we transiently co-transfected cells with MMP-9 promoter-luciferase constructs and WT-Nrf2 vectors. The transient overexpression of Nrf2 resulted in a clear enhancement of MMP-9 promoter activity (Fig. 3C, lower panel). In addition, our experiments with mutated versions of the ARE1/2 and NF-κB binding sites also showed that the MMP-9 promoter is activated by Nrf2 through the ARE1 and ARE2 binding sites, but not via the NF-κB binding site (Fig. 3D). The glucose starvation-enhanced MMP-9 promoter activity was also largely abolished using a mutated construct of the ARE1/2 (Fig. 3E). We also found that the expression of HO-1, an Nrf2-target gene, was induced along with MMP-9 expression in cells transfected with WT-Nrf2 even in normal culture conditions (Fig. S4A,B). Furthermore, expression of Nrf2 shRNA was found to diminish the enhanced MMP-9 promoter activity induced by glucose starvation (Fig. 3F). Besides this, experiments in constitutive Nrf2 expressing cell lines indicate that expression of MMP-9, and two Nrf2-regulated proteins, HO-1 and NQO1, were depressed by Nrf2 shRNA, but did not affect levels of MMP-2 expression (Fig. S4C). Taken together, these results clearly suggest that glucose starvation-induced Nrf2 expression is involved in the transactivation of MMP-9 expression via the AREs within the promoter region of the MMP-9 gene.

Next, we examined the LKB1- and AMPK-mediated activity of the MMP-9 promoter under glucose starvation conditions. We found that the enhanced transactivation of MMP-9 gene expression due to glucose starvation was inhibited by knocking down LKB1 or AMPK activity (Fig. 3G). We further confirmed that significant induction of MMP-9 mRNA expression by glucose starvation was also completely diminished by LKB1, AMPK, or Nrf2 shRNA (Fig. 3H). Interestingly, no significant Nrf2 induction within the nucleus was observed under glucose starvation in a time- and dose-dependent manner in the LKB1-deficient HeLa cell line (Fig. S5A,B); however, treatment of these cells with an exogenous chemical oxidant, tBH, or an Nrf2 inducer, tBHQ, could induce Nrf2 expression (Fig. S5C). Therefore, it is likely that Nrf2 is regulated and induced by the LKB1-AMPK signalling pathway, especially in response to glucose starvation conditions. On the basis of these results, we conclude that the LKB1-AMPK signalling pathway plays a critical role in the Nrf2-regulated transactivation of MMP-9 gene expression during conditions of energy stress.

### Glucose starvation-induced Nrf2 expression is regulated by LKB1-AMPK-mediated selective autophagy implementation

**Figure 4:**
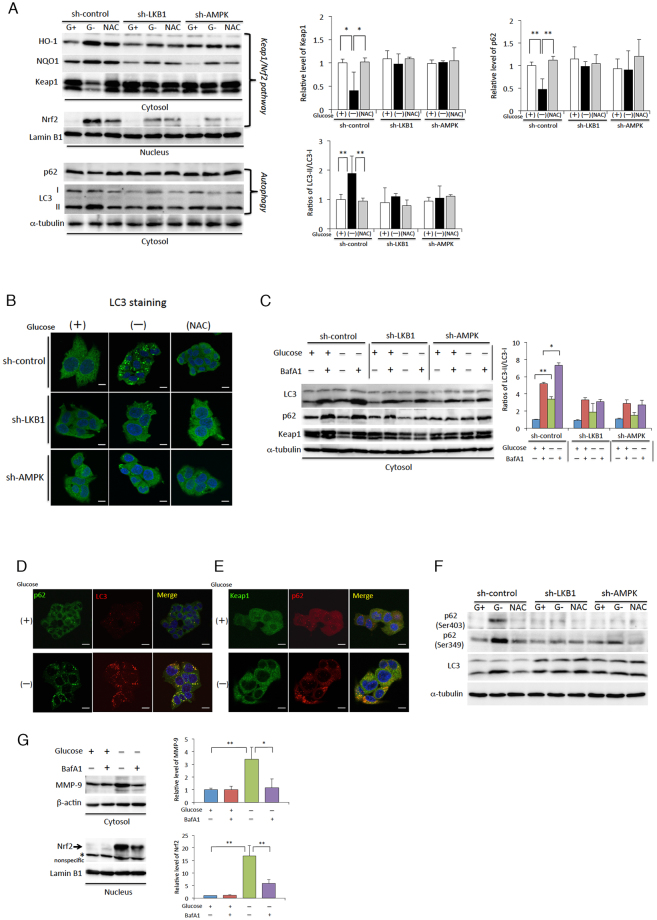
Glucose starvation regulates selective autophagy-mediated Nrf2 induction through the activation of the LKB1-AMPK signalling pathway. (**A**) HepG2 cells stably expressing sh-control, sh-LKB1, or sh-AMPK were incubated in glucose-free medium in the absence (−) or presence (+) of 5.5 mM glucose, or 2.5 mM NAC for 4 h. Western blot analysis was performed with antibodies against the indicated proteins, with either α-tubulin or Lamin B1 as loading controls. (**B**) Cellular localisation of LC3 was determined by immunofluorescence staining in cells lines expressing sh-control, sh-LKB1, or sh-AMPK after incubation in glucose-free medium in the absence (−) or presence (+) of glucose, or 2.5 mM NAC for 4 h. (**C**) Cells expressing sh-control, sh-LKB1, or sh-AMPK were cultured in glucose-free medium (−) or control medium (+) for 6 h in the presence or absence of bafilomycin A1 (BafA1). Western blot analysis was performed on protein extracts of these cells with antibodies against the indicated proteins with α-tubulin as a loading control. (**D**, **E**) Immunofluorescence staining of cells incubated in glucose-free medium in the absence (−) or presence (+) of glucose for 4 h, with antibodies against p62, LC3, or Keap1. (**F**) Western blot analysis to measure phosphorylated p62 (Ser403 or Ser349) and LC3 levels in the indicated cell lines that were cultured in the absence (G−) or presence (G+) of 5.5 mM glucose, or 2.5 mM NAC for 4 h. (**G**) HepG2 cells were cultured in glucose-free medium (−) or control medium (+) for 6 h in the presence or absence of bafilomycin A1 (BafA1). Western blot analysis was performed on protein extracts of these cells with antibodies against the indicated proteins with either β-actin or Lamin B1 as loading controls. The asterisk indicates a nonspecific band. The expression levels of Keap1, p62, MMP-9, or Nrf2 and the ratios of LC3-II/LC-I were measured by densitometric analysis. For all experiments, the data are presented as mean ± SD of 3 independent experiments. *P < 0.05; **P < 0.01 Uncropped blots of all experiments are presented in Supplementary Fig. 9. The scale bar in all microscope images represents 10 μm.

Autophagy is a catabolic process aimed at recycling cellular components and damaged organelles in response to diverse conditions of cellular stress, such as nutrient deprivation, viral infection, or genotoxic damage^34,35^. It has been widely proven that the LKB1-AMPK signal activated during glucose starvation conditions also activates starvation-induced autophagy^36,37^. Besides this, LKB1-AMPK-induced autophagy has also been shown to mediate resistance to oxidative stress, though the mechanistic details of this process are not clear enough^34^. Therefore, we investigated if LKB1 and/or AMPK are involved in the induction of Nrf2 gene expression via glucose starvation-induced autophagy. As expected, under glucose starvation conditions, levels of the autophagosome marker, LC3, were increased, along with a decrease in the levels of p62/SQSTM1 protein (hereafter referred to as p62); expression levels of nuclear Nrf2, HO-1, and NQO1 were also greatly increased, and treatment with NAC significantly inhibited these changes in the sh-control cells (Fig. 4A). Furthermore, not only the glucose starvation-induced expression, but also abundance of cells staining positive for LC3 were highly reduced by NAC treatment and by expression of LKB1 or AMPK shRNA (Fig. 4B). In contrast to these results, the LKB1-AMPK-mediated pathway had little direct effect on amino acid deprivation-meditated autophagy (Fig. S6A,B).

Following these experiments, we performed LC3 flux analysis using bafilomycin A1^38^, an inhibitor of the late phase of autophagy to investigate the role of autophagy in the induction of Nrf2 expression. Defects in autophagy flux caused by knockdown of LKB1 or AMPK were confirmed by western blot analysis, which provides a simultaneous readout for autophagosome formation and maturation (Fig. 4C). Intriguingly, the expression of an Nrf2 regulator, Kelch-like ECH-associated protein 1 (Keap1), was markedly decreased by glucose starvation, in a pattern similar to that of p62 expression; this reduction in Keap1 and p62 levels was prevented by NAC treatment and expression of LKB1 or AMPK shRNA (Fig. 4A). The flux analysis using bafilomycin A1 indicated that Keap1 expression was also regulated by glucose starvation-induced autophagy (Fig. 4C). In addition, we also observed that p62 is not only colocalised with LC3, but also with Keap1 under glucose starvation (Fig. 4D,E). These findings suggested that Keap1 expression is regulated by selective autophagy. We also found that glucose starvation induced phosphorylation of p62 at Ser349 (Fig. 4F). Since phosphorylation of p62 at Ser349 competitively abrogates the interaction between Keap1 and Nrf2, following which, the Keap1 in a complex with phosphorylated p62 is degraded by selective autophagy, this step is essential in facilitating the translocation of Nrf2 into the nucleus^39^. Furthermore, although the activation of the LKB1-AMPK signal is of crucial importance in the phosphorylation of p62 at Ser349, phosphorylation at Ser403 was also observed in response to glucose starvation-mediated autophagy; the phosphorylation at Ser403 was also inhibited by either NAC treatment, or knockdown of LKB1 or AMPK (Fig. 4F). These observations and their relationships to the decrease in Keap1 were not detected under amino acid starvation conditions (Fig. S6C), suggesting that p62 phosphorylation-mediated selective autophagy is a specific event in the adaptation response to glucose starvation stress. As p62 is also required for ubiquitination-dependent mitochondrial autophagy (mitophagy)^40,41^, it was unsurprising that we also found p62 colocalizing with the mitochondrial outer-membrane protein TOM20 and ubiquitin under glucose starvation conditions (Fig. S7A,B). Glucose starvation caused slight reductions in the levels of TOM20 and the inner-membrane protein TIM23; these changes were suppressed by NAC treatment and expression of LKB1 or AMPK shRNA (Fig. S7C). Furthermore, we also have experimental data that suggests a link between MMP-9 and Nrf2 induction and autophagy during glucose starvation. Glucose starvation-induced MMP-9 and Nrf2 expression were strongly inhibited when the autophagy maturation process is inhibited using bafilomycin A1 (Fig. 4G). Taken together, our observations strongly suggest that LKB1-AMPK activation is not only involved in autophagy during glucose starvation, but also regulates autophagy-mediated maintenance of redox balance in cells, all of which integrate into the mechanism that leads to the induction of MMP-9 expression to help cells escape from severe metabolic stress.

### LKB1-AMPK-mediated autophagy and not mTOR-mediated autophagy is required for the phosphorylation of p62 and induction of MMP-9 expression under glucose starvation conditions

**Figure 5:**
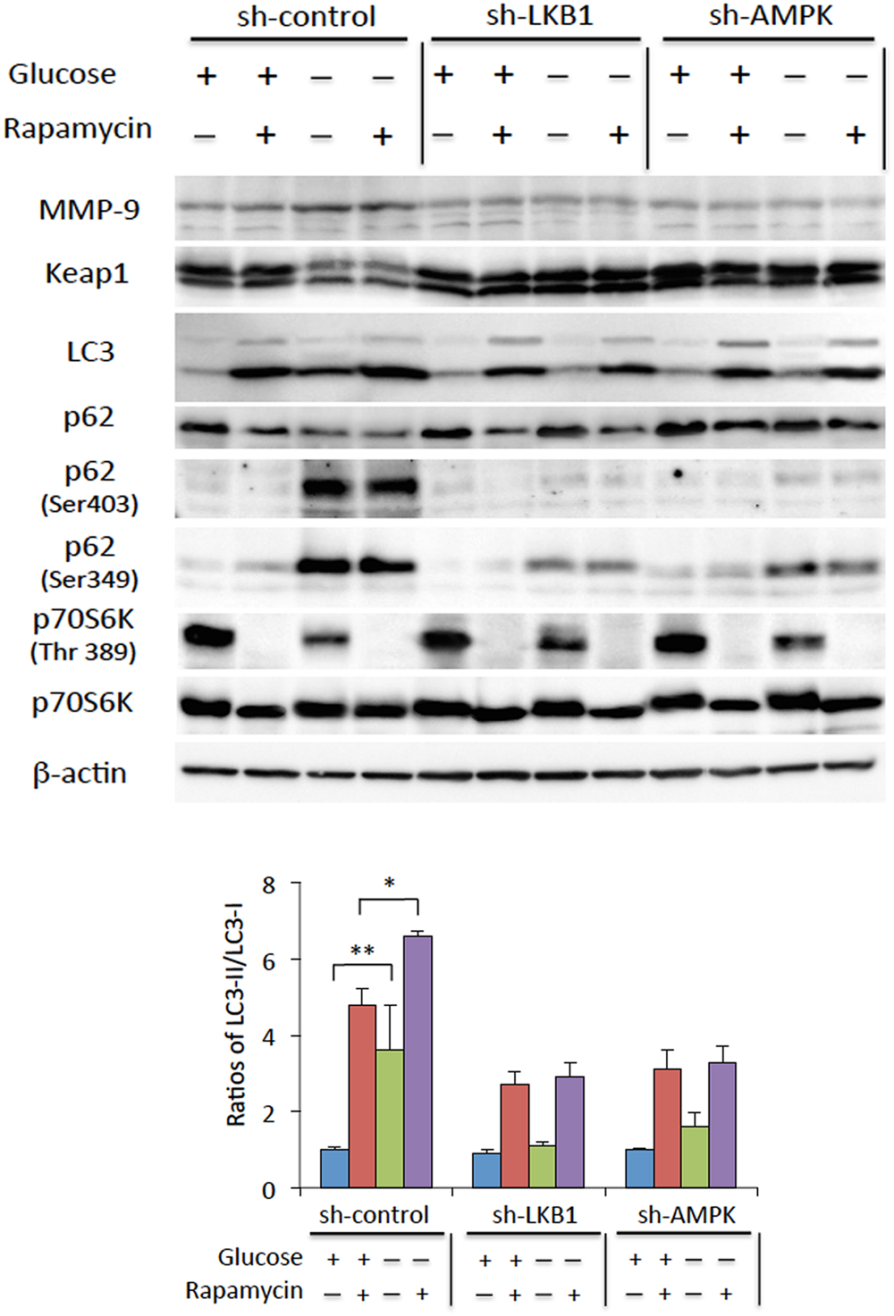
Phosphorylation of p62 is induced by activation of the LKB1-AMPK signalling pathway, but is not dependent on or regulated by mTORC1. HepG2 cells expressing sh-control, sh-LKB1, or sh-AMPK were cultured in glucose-free medium (−) or control medium (+) for 6 h in the presence or absence of rapamycin, and whole-cell lysates were analysed by western blotting with antibodies against the indicated proteins. The ratios of LC3-II/LC-I were measured by densitometric analysis. All the data shown here are representative of 3 independent sets of experiments. Uncropped blots of all experiments are presented in Supplementary Fig. 9.

It is well known that autophagy is inhibited by the central cell-growth regulator, mTORC1^42^, and that AMPK stimulates autophagy by inhibiting mTORC1 activation^43^. We found that cells treated with the specific inhibitor of mTOR kinase, rapamycin, exhibited increase in ratios of LC3-II/I and reduction in p62 levels, both, in the presence and absence of glucose, indicating that autophagy was induced irrespective of glucose availability (Fig. 5). Inhibition of the mTORC1 pathway was monitored by monitoring the decrease in phosphorylation of p70S6K at Thr389, a specific mTORC1 substrate; this phenomenon was observed to occur under glucose starvation conditions (Fig. 5). Exposure to rapamycin did not seem to affect glucose starvation-induced p62 phosphorylation and suppression of Keap1 expression, or induction of MMP-9 expression (Fig. 5). As several kinases other than mTORC1 phosphorylate p62 for selective autophagy mechanisms^44,45^, our observations indicate that glucose starvation-induced Nrf2 and MMP-9 expression are probably regulated by an mTORC1-independent autophagy pathway. Furthermore, we observed that rapamycin treatment did not rescue the loss of glucose starvation-induced p62 phosphorylation (Fig. 5). These results indicate quite clearly that nonselective autophagy dependent on the mTOR signal is dispensable for the induction of MMP-9 expression under glucose starvation conditions.

### LKB1-AMPK signal is essential for glucose starvation-induced MMP-9 expression through an integrative set of mechanisms involving Nrf2 induction

**Figure 6:**
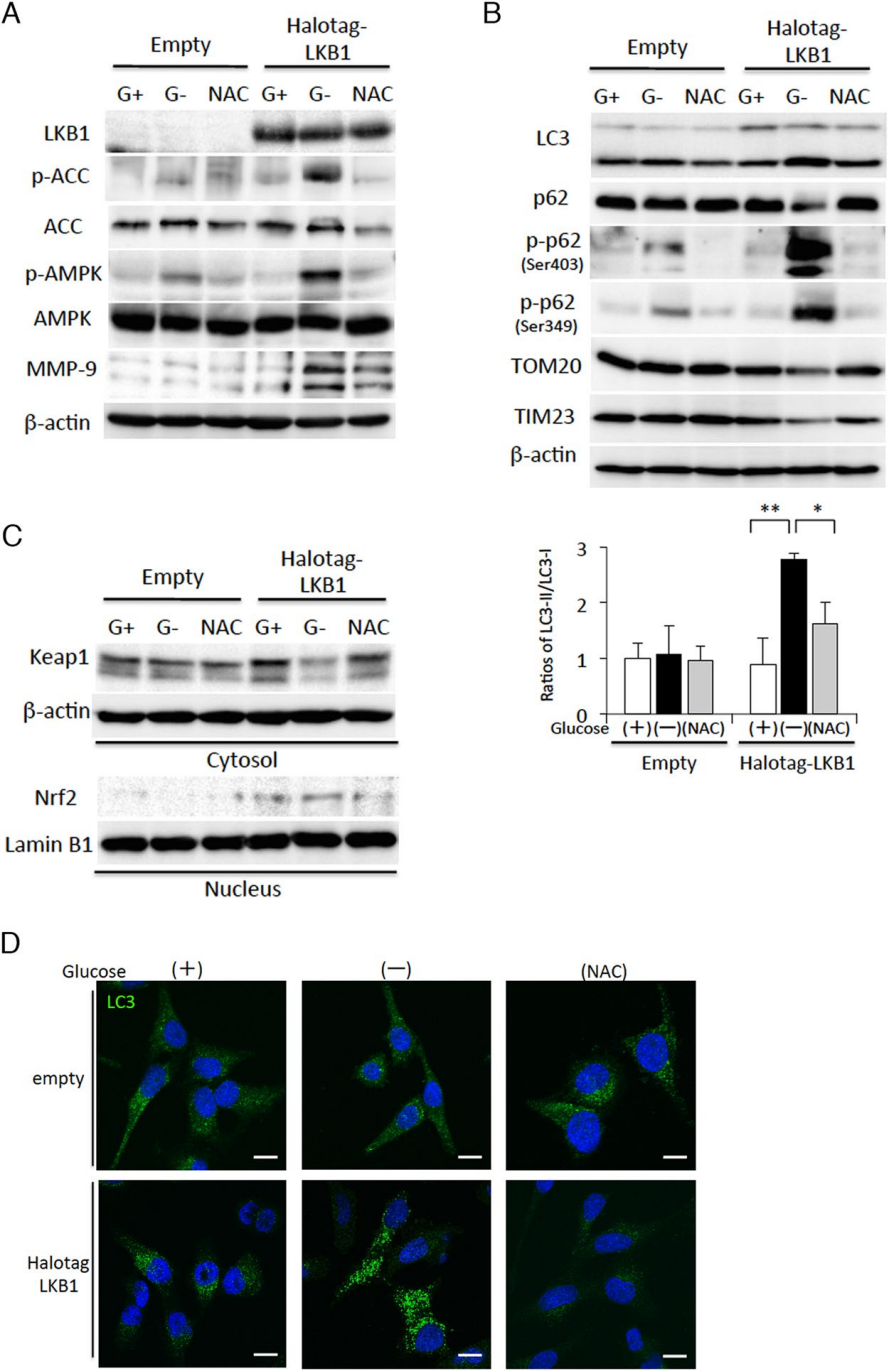
Activation of the LKB1-AMPK signalling pathway is required for glucose starvation-mediated induction of MMP-9 expression via oxidative stress responses. (**A**–**C**) HepG2 cells stably expressing empty control vector or Halotag-fused LKB1 (Halotag-LKB1) were incubated in glucose-free medium in the absence (G−) or presence (G+) of 5.5 mM glucose, or 2.5 mM NAC for 4 h. Western blot analysis was performed with antibodies against the indicated proteins with either β-actin or Lamin B1 as loading controls. The ratios of LC3-II/LC-I were measured by densitometric analysis. (**D**) Cellular localisation of LC3 was determined by immunofluorescence staining in cell lines expressing empty control vector or Halotag-LKB1 vector after incubation in glucose-free medium in the absence (−) or presence (+) of glucose, or 2.5 mM NAC for 4 h. The scale bar represents 10 μm. All the data shown here are representative of 3 independent sets of experiments. Uncropped blots of all experiments are presented in Supplementary Fig. 9.

To further confirm that the LKB1-AMPK signalling pathway plays an essential role in glucose starvation-mediated MMP-9 expression, we established stable lines of LKB1-expressing HeLa cells following transfection with Halo-tagged-LKB1 (Halotag-LKB1); HeLa cells transfected with the empty vector served as a negative control. LKB1 reconstitution in HeLa cells restored AMPK activation, as indicated by ACC phosphorylation, and the induction of MMP-9 expression under glucose starvation conditions; all these phenomena were inhibited by NAC treatment (Fig. 6A). Furthermore, reconstitution of LKB1 expression also promoted selective autophagy-induced p62 phosphorylation, and reduced levels of TOM20, TIM23, and Keap1 (Fig. 6B), which, in consequence, also led to rising levels of nuclear Nrf2 (Fig. 6C). All these phenomena following LKB1 reconstitution were inhibited by NAC treatment (Fig. 6A–C). Furthermore, treatment of the HeLa cells with reconstituted LKB1 activity with compound C, a specific inhibitor of AMPK activity, completely inhibited the induction of LC3, Nrf2, and MMP-9 expression during glucose starvation (Fig. S8). These results clearly indicate that the LKB1-AMPK pathway drives the induction of MMP-9 expression through an integrative mechanism involving antioxidant responses and selective autophagy-mediated Nrf2 expression.

## Discussion

Cancer cells often have huge energy requirements in order to grow and divide rapidly, and it is well known that cancer cells rely heavily on the glycolytic pathway to produce energy and metabolites for growth even under conditions of sufficient oxygen availability^1–3^. However, limited nutrient availability is one of the most distinguishing characteristics of the cancer microenvironment. This condition is not only caused by the high nutrient demands of cancer cells, but also due to the fact that most solid tumours tend to outgrow existing vasculature; cells that survive such conditions eventually gain highly malignant characteristics.

Although the development of malignant characteristics in the cells involved in cancer progression are not entirely explained by their ability to survive such gruelling energy-depleted conditions, it is likely that these conditions strongly affect the development of malignant phenotypes. In our study, we have shown a novel integrative molecular mechanism by which the intracellular metabolic sensor LKB1-AMPK regulates molecular events central to cancer cell migration and invasiveness under glucose starvation conditions. Our work shows that under glucose starvation conditions, oxidative stress-induced LKB1-AMPK activation regulates transactivation of MMP-9 through selective autophagy-mediated Nrf2 induction, and that this sequence of events can contribute to cell survival and aid cancer cells in escaping such nutrient-starved conditions. Therefore, our results have uncovered a critical role played by the LKB1-AMPK signal that could serve as a base for metabolic adaptation in cells under severe microenvironmental stress. Our findings could also explain why strong microenvironmental stresses can cause the development of aggressive characteristics in tumour cells.

Although the LKB1-AMPK signalling pathway is known to be a key signalling pathway essential for maintaining intracellular energy levels especially in response to low-nutrient conditions such as glucose starvation^24,46,47^, its other roles in regulating energy homeostasis, especially in tumour cells, are not fully understood. As the role of LKB1 as a tumour suppressor has been well documented, AMPK is also considered to be a tumour suppressor due to its function as a component of the LKB1-mediated tumour suppressor cascade^8,11^. On the other hand, both LKB1- and AMPK-null mouse embryonic fibroblasts (MEFs) are resistant to H-Ras^V12^-induced anchorage-independent growth, as well as solid tumour growth *in vivo*^12,13^. Furthermore, AMPK has been shown to be essential for oncogene-induced tumour development and cell proliferation processes in some cancers, as well as for anoikis resistance in MEFs and certain cancer cell lines^14,48,49^. During cell migration and invasion, pharmacological activation of AMPK leads to mitochondrial trafficking, and changes in ATP content and cytoskeletal dynamics^50^. Moreover, it has been shown that the LKB1-AMPK signalling pathway is required for cancer cell survival and spheroid migration under low-nutrient conditions^49,51^. Therefore, the role of the LKB1-AMPK signal in cancer progression, especially during conditions of nutrient deprivation, remains controversial.

In our study, we have clearly shown that the LKB1-AMPK signalling pathway contributes to cancer cell migration and invasiveness by induction of MMP-9 expression via an Nrf2-induced pathway that is activated in response to ROS produced under conditions of glucose starvation. Under severe microenvironmental stresses, ROS levels must be managed stringently for cells to survive and develop invasive and metastatic characteristics^18^. This is because although ROS levels are essential for cancer progression, excess ROS can cause cell death by oxidative stress. We find that during glucose starvation, LKB1-AMPK-mediated Nrf2 induction not only induces the expression of its target antioxidant enzymes HO-1 and NQO1, but also induces MMP-9 expression, which could be a two-pronged strategy for preventing the buildup of high ROS levels, as well as to escape from a hostile microenvironment. Furthermore, adequately controlled ROS levels function in signalling networks involved in numerous pathways that regulate cellular metabolism, epithelial-mesenchymal transition, autophagy, and cell survival in cancer cells^18,34,52^. Inhibition of LKB1 and AMPK in cells under glucose starvation caused excess intracellular ROS to be generated, while Nrf2 induction and selective autophagy implementation failed to occur. In glucose-starved cells with functional LKB1-AMPK, treatment with an ROS scavenger, NAC, diminished the LKB1-AMPK signal, suggesting that the antioxidant system regulated through autophagy induction is essential for the proper functioning of the LKB1-AMPK signal in helping cells to adapt to microenvironmental stress. Recently, a report by Jeon *et al*. indicates that the LKB1-AMPK pathway contributes significantly to the regulation of metabolic homeostasis essential for cancer cell survival^24^. This is because AMPK activation is required to maintain intracellular NADPH levels in response to metabolic stress, and NADPH is the major reducing agent used in regenerating oxidised glutathione and thioredoxin, both of which scavenge ROS during oxidative stress^53^. Intriguingly, most genes coding for enzymes involved in NADPH synthesis, such as glucose-6-phosphate dehydrogenase (G6PD), malic enzyme 1 (ME1), and isocitrate dehydrogenase 1 (IDH1), are targeted by Nrf2^54^. Our study clearly shows that LKB1-AMPK activation induces Nrf2 under glucose starvation, which means that these Nrf2-targeted enzymes are in turn regulated by the LKB1-AMPK pathway; this emphasises the importance of LKB1-AMPK in maintaining cellular energy homeostasis and redox balance under glucose starvation.

Our findings also indicate that the LKB1-AMPK signal plays a prominent role in the induction of selective autophagy, which regulates the accumulation of Nrf2 in the nucleus and transactivation of MMP-9 under glucose starvation. This process involves the selective autophagy receptor, p62 which has multiple domains that mediate its interactions with various binding partners^44,45^, for degradation of ubiquitinated substrates^41^. The phosphorylation of p62 at Ser403 by casein kinase 2 or TANK-binding kinase 1 reportedly increases the affinity of p62 for polyubiquitinated proteins, and enhances these proteins’ autophagic degradation^40,55^. One study further reports that mTORC1-dependent phosphorylation of p62 at Ser351 (Ser349 in humans) increases the binding affinity of p62 for Keap1, and competitively inhibits the Keap1-Nrf2 interaction^39^. As Keap1 in complex with phosphorylated p62 is degraded by selective autophagy, Nrf2 expression is stabilised even under nutrient-rich conditions^39^. Since AMPK is involved in the induction of autophagy through inhibition of mTORC1, under nutrient limited conditions^42,43^, we also investigated if mTORC1-induced autophagy was important for p62 phosphorylation and Nrf2 induction. However, our results indicate that mTORC1-induced autophagy is not necessary for LKB1-AMPK-induced Nrf2 activity or MMP-9 expression.

**Figure 7:**
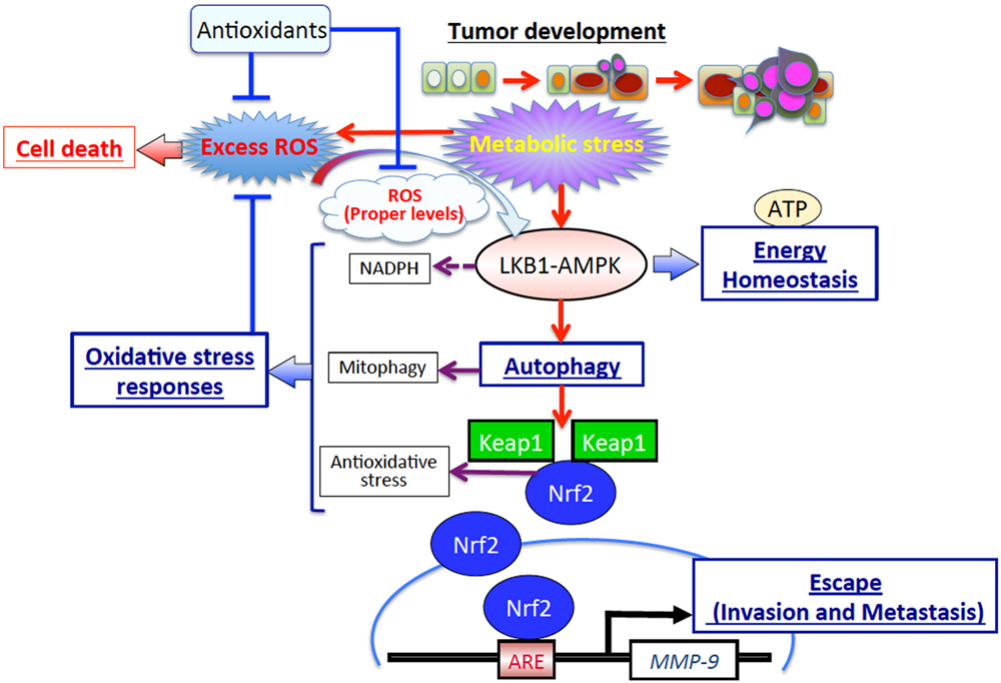
A schematic representation of the hypothetical model proposed by us outlining the role of the LKB1-AMPK signalling pathway in activating metabolic stress-mediated MMP-9 expression during cancer progression. The schematic shows that the LKB1-AMPK signalling pathway induces the expression of MMP-9 through an integrated network of signals. Metabolic stresses such as glucose starvation initiate LKB1-AMPK signalling via the generation of controlled levels of ROS, to eventually affect a host of different intracellular functions such as energy metabolism, autophagy, oxidative stress response, and cellular motility to establish metabolic adaptation and cancer progression.

Growing evidence indicates that somatic mutations identified in Keap1 and Nrf2 occur in clinical samples of diverse types of cancers^56–58^. These mutations result in the disruption or alteration of the Keap1-Nrf2 interaction and lead to persistent and constitutive activation of Nrf2. Mitsuishi *et al*.^54^ have shown that constitutive upregulation of Nrf2 orchestrates profound metabolic changes by directing glucose and glutamine into anabolic pathways, and that this alteration contributes significantly to cell proliferation^54^. Despite the powerful effects exerted by these somatic mutations in Keap1 or Nrf2 that lead to the constitutive activation of Nrf2, such mutations were only found in ~1.45% of all cancer samples examined in studies published in COSMIC 2018 (COSMIC; http:// cancer.sanger.ac.uk/cosmic). Therefore, it seems likely that the constitutive activation of Nrf2 is not the most important step in cancer development, but that other mechanisms—such as the LKB1-AMPK signalling pathway—that allow tightly regulated and appropriate Nrf2 induction while maintaining cellular homeostasis under microenvironmental stresses are also required. Our data indicate that the LKB1-AMPK signalling pathway plays an important role in cellular adaptation to microenvironmental conditions. The LKB1-AMPK signalling pathway is central to an integrative molecular network that results in the induction of MMP-9 expression; this is accomplished by Nrf2 activation by a selective autophagy pathway that contributes to the survival of cells and progression of cancer (Fig. 7). Notably, our observations reveal a new mechanism where glucose starvation-induced LKB1-AMPK activation functions as a starting point to regulate energy homeostasis, autophagy, and oxidative stress responses, to induce MMP-9 expression. We believe that our findings have potential in facilitating the development of therapeutic strategies targeting metabolic adaptation mechanisms in various cancer types.

## Materials and Methods

### Reagents and antibodies

All chemical and biochemical reagents were obtained from the following sources: D-glucose, L-glutamine, and mitomycin C were purchased from Wako Pure Chemical (Osaka, Japan); compound C and Rapamycin were from Calbiochem-Merck (Darmstadt, Germany); Bafilomycin A1, Earle’s Buffered Salt Solution (EBSS), Dulbecco’s modified Eagle’s medium (DMEM), dimethylsulfoxide (DMSO), MEM amino acids solution (MEM EAA), N-acetyl-L-cysteine (NAC), proteinase or phosphatase inhibitor cocktail, puromycin, *tert*-butyl hydroperoxide (tBH), and *tert*-butyl hydroquinone (tBHQ) were from Sigma (St. Louis, MO, USA). Antibodies against AMPKα, p-AMPKα (Thr172), ACC, p-ACC (Ser79), IDH1, LKB1, PARP, p-p62 (Ser349), p70S6K, p-p70S6K (Thr389), Raptor, p-Raptor (Ser792), and Ubiquitin were obtained from Cell Signalling (Beverly, MA). Antibodies against α-tubulin, β-actin, and MMP-9 were purchased from Sigma. Antibodies against lamin B1 and LC3 were from MBL (Nagoya, Japan); antibodies against Keap1 and NQO1 were from Proteintech group (Chicago, IL). Antibodies against HO-1, TIM23, TOM20, and MMP-13 were from Enzo Life Sciences (Plymouth Meeting, PA), BD Biosciences (San Jose, CA), Santa Cruz Biotechnology (Santa Cruz, CA), and Daiichi Fine Chemicals (Takaoka, Japan), respectively. Antibodies against G6PD, p62, ME1, and MMP-2 were from Abcam (Cambridge, UK), and antibodies against p-p62 (Ser403) and Nrf2 were from GeneTex (San Antonio, TX)

### Cell cultures and treatments

A549, HeLa, BxPC-3, Panc-1, HepG2, and Huh-7 cells were grown in DMEM supplemented with 10% fetal bovine serum (FBS), 50 U/mL penicillin, 50 mg/mL streptomycin, and non-essential amino acids (Life Technologies, Carlsbad, CA) at 37 °C and 5% CO_2_. For glucose starvation experiments, cells were washed with PBS and cultured to 70–80% confluence in glucose-free DMEM containing 10% dialysed FBS (Biological Industries, Beit Haemek, Israel). For amino acid starvation experiments, cells were rinsed and treated with EBSS containing 10% dialysed FBS.

### Plasmids and stable transfections

HepG2 cells were transfected with MISSION® short hairpin targeting human LKB1 (sh-LKB1; CCGGGCCAACGTGAAGAAGGAAATTCTCGAGAATTTCCTTCTTCACGTTGGCTTTTT) containing plasmid, human AMPKα1 (sh-AMPK; CCGGCCATCCTGAAAGAGTACCATTCTCGAGAATGGTACTCTTTCAGGATGGTTTTT) containing plasmid (both from Sigma), or SureSilencing shRNA plasmid for targeting human NFE2L2/Nrf2 (sh-Nrf2; SABioscience, Frederick, MD, USA) using Lipofectamine LTX Reagent with PLUS Reagent (ThermoFisher Scientific, Waltham, MA) for 48 h. Non-targeting control shRNA plasmid served as a negative control (sh-control). Cells were then transferred into 2 μg/mL puromycin-containing medium for 3 weeks for single-cell clone selection to obtain a stable expression cell line. The pFN21A clone expressing an N-terminal HaloTag fusion of human full-length LKB1 (HaloTag-LKB1) was obtained from Kazusa DNA Research Institute (Kisarazu, Japan). HaloTag empty vector (Promega, Madison, WI, USA) was used for expression of the HaloTag protein alone and served as a control. To develop stable expression cell lines, HeLa cells, which do not express endogenous LKB1, were transfected with either the HaloTag empty vector or the Halotag-LKB1 construct, and were cultured in the presence of 850 μg/mL Geneticin (G418; Sigma).

### Luciferase assays to test for MMP-9 promoter activity

Wild-type (WT) and mNF-κB (a mutated version of the NF-κB binding site) constructs of the human MMP-9 promoter cloned into the pGL3 basic vector (Promega) were kindly provided by Dr. Thomas Iftner (University Hospital of Tubingen, Tubingen, Germany)^59^. Introduction of point mutations into the distal ARE (mARE1), the proximal ARE (mARE2), and multiple ARE (mARE1/2) were made with PrimeSTAR Mutagenesis Basal Kit (TaKaRa Bio, Shiga, Japan) according to the manufacturer’s instructions; the presence of these mutations were confirmed by DNA sequencing. The wild-type NRF2 expression vector (WT-Nrf2) was a gift from H.S. So (Wonkwang University School of Medicine, Korea)^60^. HepG2 cells were grown in in 6-well plates to 80% confluence before transfection with reporter plasmids. Cells were transfected with a total of 2 μg each of the luciferase reporter constructs using LipofectAMINE plus (Life Technologies). To ensure efficient transfection, Renilla luciferase gene expression was monitored using either the pRL-CMV or pRL-TK vectors (Promega). Thirty-six hours after transfection, the cells were transferred to and maintained in glucose-depleted medium for 6 h, following which, they were harvested, and luciferase activity in the cells was analysed using a Dual-Luciferase Reporter Assay System (Promega). For experiments requiring transient overexpression of 0.5 μg WT-Nrf2, sh-LKB1, sh-AMPK, or sh-Nrf2, the total plasmid concentration used for transfection was normalised using the pcDNA3.1(+) empty vector or sh-control vector.

### Western blot analysis

Western blot analysis was performed as described previously^61^. Nuclear and cytoplasmic proteins were extracted using the NE-PER nuclear and cytoplasmic extraction kit (ThermoFisher Scientific) according to the manufacturer’s protocol. For protein extraction, the cells were lysed in a radioimmunoprecipitation assay (RIPA) buffer (Sigma) containing complete protease and phosphatase inhibitor cocktail. Equal amounts of protein were then resolved by sodium dodecyl sulfate-polyacrylamide gel electrophoresis (SDS-PAGE) and transferred to a polyvinylidene difluoride (PVDF) membrane. The membranes were developed by chemiluminescence using Immobilon Western Chemiluminescent HRP Substrate (Millipore, Billerica, MA).

### Immunofluorescence assays

Cells were plated onto 4-well chamber slides for 24 h, following which, the medium was replaced with glucose-free medium either with or without glucose. After 6 h, the cells were fixed with 4% paraformaldehyde in PBS for 10 min and washed with PBS. Cells were permeabilised with 0.1% Triton X-100 for 5 min and washed with PBS before the addition of and incubation in 5% BSA for 30 min. Antibodies were diluted in 0.1% BSA in PBS and incubated with the cells overnight at 4 °C. Slides were embedded with ProLong Gold anti-fade reagent (Life Technologies) and confocal images were obtained using an LSM 700 confocal microscope (Carl Zeiss, Jena, Germany) at a magnification of 63X.

### Determination of ATP content

Intracellular ATP levels were determined by luminescence using the CellTiter-Glo 2.0 Luminescent Cell Viability Assay (Promega) as per the manufacture’s instruction. Briefly, 1 × 10^4^ cells/100 μL were loaded into each well in a 96-well plate. After addition of 100 μL volumes of CellTiter-Glo reagents, relative luminescence units were measured using GloMax 96 microplate luminometer (Promega) and expressed as fold changes. All values were normalised to protein concentrations.

### ROS assays

The ROS-Glo H_2_O_2_ assay (Promega) was used to measure the level of hydrogen peroxide (H_2_O_2_) in culture according to the manufacturer’s instructions. The ROS assay was carried out by plating 1 × 10^4^ cells/100 μL into each well in a 96-well plate. Cells were incubated with or without glucose and H_2_O_2_ substrate solution for 4 h, following which the ROS-Glo detection solution was added. Luminescence units were measured using GloMax 96 microplate luminometer (Promega). All the values were normalised to protein concentrations.

### Wound healing assays

Cells plated into a 6-well plate at 90% confluence were pre-treated with mitomycin C (2 μg/mL) for 3 h to inhibit proliferation, following which, an injury line of approximately 2 mm was made with a sterile scraper across the cell monolayer. The floating cell debris was removed by washing with PBS, after which, cells were incubated for 0 or 24 h in the presence of specific glucose concentrations with mitomycin C. Cell migration was monitored by phase-contrast microscopy using a Nikon Diaphot 300 (Nikon, Tokyo, Japan).

### Transwell matrigel invasion assay

Invasion assays were carried out using Matrigel invasion chambers with membranes of 8 μm pore size (Corning, NY, USA). The upper compartments were seeded with 1 × 10^5^ cells in DMEM containing specific glucose concentrations. DMEM containing 10% FBS as a chemo-attractant was added to the lower compartments. After a 24 h incubation period, non-invading cells were removed from the upper surface of the membrane by scrubbing with cotton tipped swabs. The invasive cells on the membranes beneath the insert were fixed with ice-cold methanol and stained using the Diff-Quick staining kit (Dade-Behring, Newark, DE) according to the manufacturer’s instructions. The stained cells were counted using BX63 light microscope (Olympus, Tokyo, Japan). Five randomly selected fields were chosen, and the total number of cells in each field was counted, following which, an average was obtained to provide a measure for the number of invasive cells in this assay.

### Cell proliferation assay

For cell proliferation assays, the CellTiter 96 AQueous One Solution Cell Proliferation Assay system (Promega) was used according to the manufacturer’s instructions. 5 × 10^3^ cells were plated into each well in a 96-well and 10 μL per well of CellTiter 96 AQueous One Solution reagent was added. After 1 h incubation in humidified 5% CO_2_ atmosphere, absorbance at 490 nm was measured every 24 h using a SpectraMax I3 microplate reader (Molecular Devices, Sunnyvale, CA). Six replicate wells per time point were used to obtain measures of cell proliferation.

### Semi-quantitative RT-PCR analysis

mRNA expression levels were determined by semi-quantitative RT-PCR analysis as described previously^62,63^. Total RNA was isolated using the GenElute Mammalian Total RNA Kit (Sigma). The specific primers used for first strand cDNA synthesis and PCR have been described previously^62,63^. PCR products were separated on a 1.8% agarose gel and stained with ethidium bromide for analysis.

### Quantitative real-time RT-PCR analysis

Quantitative real-time RT-PCR was performed using the SYBR Green PCR master mix (Applied Biosystems, Foster City, CA) with the specific primers as follows: *MMP-9* forward (5′-GCAGATTCCAAACCTTTGAG-3′), *MMP-9* reverse (5′-GCAAGTCTTCCGAGTAGT-3′), and *β-actin* forward (5′-AAAGACCTGTACGCCAACAC-3′), *β-actin* reverse (5′-GTCATACTCCTGCTTGCTGA-3′).

### Gelatin zymography

Gelatin zymography was performed as described previously^63,64^. Conditioned culture media were collected and concentrated using Amicon Ultra centrifugal filters (Millipore). Samples were mixed with SDS sample buffer without reducing agent and separated on 7.5% SDS-PAGE gels containing 0.1% gelatin (Wako). After removal of SDS by washing with 2.5% Triton X-100-containing buffer, the gels were incubated at 37 °C for 18 h to detect gelatinolytic activity. The gels were stained with Coomassie Brilliant Blue (CBB) R250, and the gelatinolytic activity in the culture media was detected as a clear band against the background of the CBB-stained gel.

### Statistical analysis

Data are expressed as the mean ± SD of measures used. Differences between treatment groups were analysed either by Student’s *t*-tests (two-tailed) or by one-way analysis of variance (ANOVA) tests. Test results with *P-values* < 0.05 are considered statistically significant.

## Electronic supplementary material

Supplementary Information
