# Supplementary material for: Glucose starvation induces LKB1-AMPK-mediated MMP-9 expression in cancer cells
Source: Sci Rep. 2018 Jul 4;8:10122. doi: 10.1038/s41598-018-28074-w (PMC6031623; doi:10.1038/s41598-018-28074-w)
Supplement: Supplementary file 1 — Supplementary Information [file 41598_2018_28074_MOESM1_ESM.docx]

**Supplementary Information**

# Glucose starvation induces LKB1-AMPK-mediated MMP-9 expression in cancer cells

Hitoshi Endo^a,*^, Satoshi Owada ^a^, Yutaka Inagaki^b^, Yukari Shida ^a^, and Masayuki Tatemichi^a^

^a^ Center for Molecular Prevention and Environmental Medicine, Department of Preventive Medicine, Tokai University School of Medicine, 143 Shimokasuya, Isehara, Kanagawa, 259-1193, Japan

^b^ Center for Matrix Biology and Medicine, Department of Regenerative Medicine, Tokai University School of Medicine, 143 Shimokasuya, Isehara, Kanagawa, 259-1193, Japan

^*^To whom correspondence may be addressed: Hitoshi Endo, Ph.D.

**Contact Information:** Hitoshi Endo, Ph.D.

Center for Molecular Prevention and Environmental Medicine, Department of Preventive Medicine, Tokai University School of Medicine, 143 Shimokasuya Isehara, Kanagawa, 259-1193, Japan

Phone: +81-463-93-1121, Fax: +81-463-92-3549

#
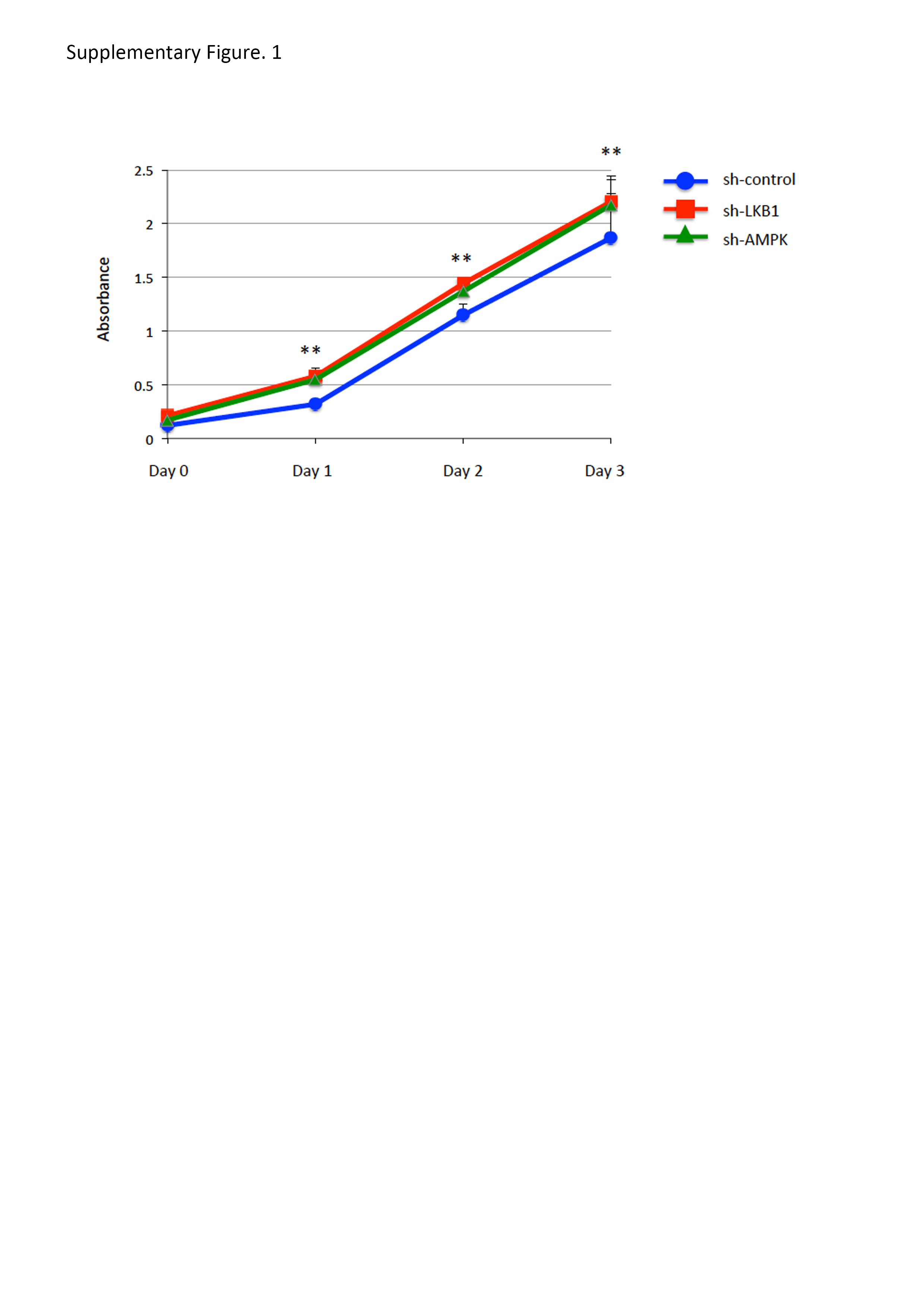
Supplementary figures and legends:

**Figure S1.** Knockdown of LKB1 or AMPK increases cell proliferation. Cell proliferation rates of HepG2 cells stably expressing sh-control, sh-LKB1, or sh-AMPK were determined by the CellTiter 96 AQueous One Solution cell proliferation assay kit under normal culture conditions. The data are presented as mean ± SD of 3 independent experiments. ** *P* < 0.01.

**
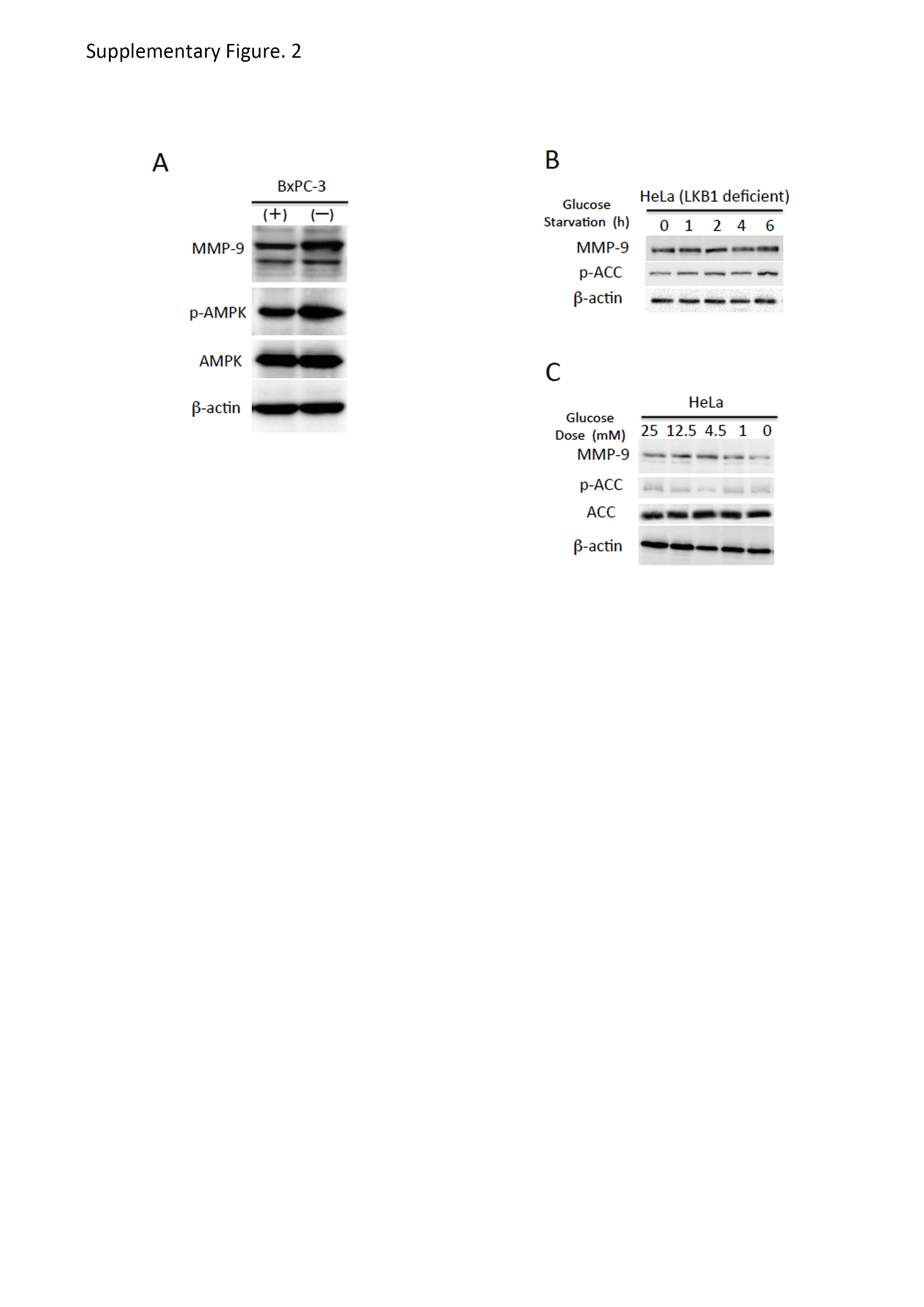
**

**Figure S2.** MMP-9 expression and AMPK activation is induced in BxPC-3 cells after prolonged exposure to glucose starvation. (A) Western blots of protein extracts obtained from BxPC-3 cells incubated in glucose-free medium in the absence (-) or presence (+) of 5.5 mM glucose for 12 h. The effects of metabolic stress conditions on MMP-9 expression in the absence of LKB1-AMPK signalling were observed using HeLa cells which are deficient in LKB1 expression. (B) HeLa cells were incubated in the absence of glucose for the indicated time periods. (C) HeLa cells were incubated in the presence of glucose of the indicated concentrations. Western blot analysis was performed with antibodies against the indicated proteins with either α-tubulin or β-actin as loading controls. All data shown here are representative of 3 independent sets of experiments. Uncropped blots for these experiments are presented in supplementary figure 9.


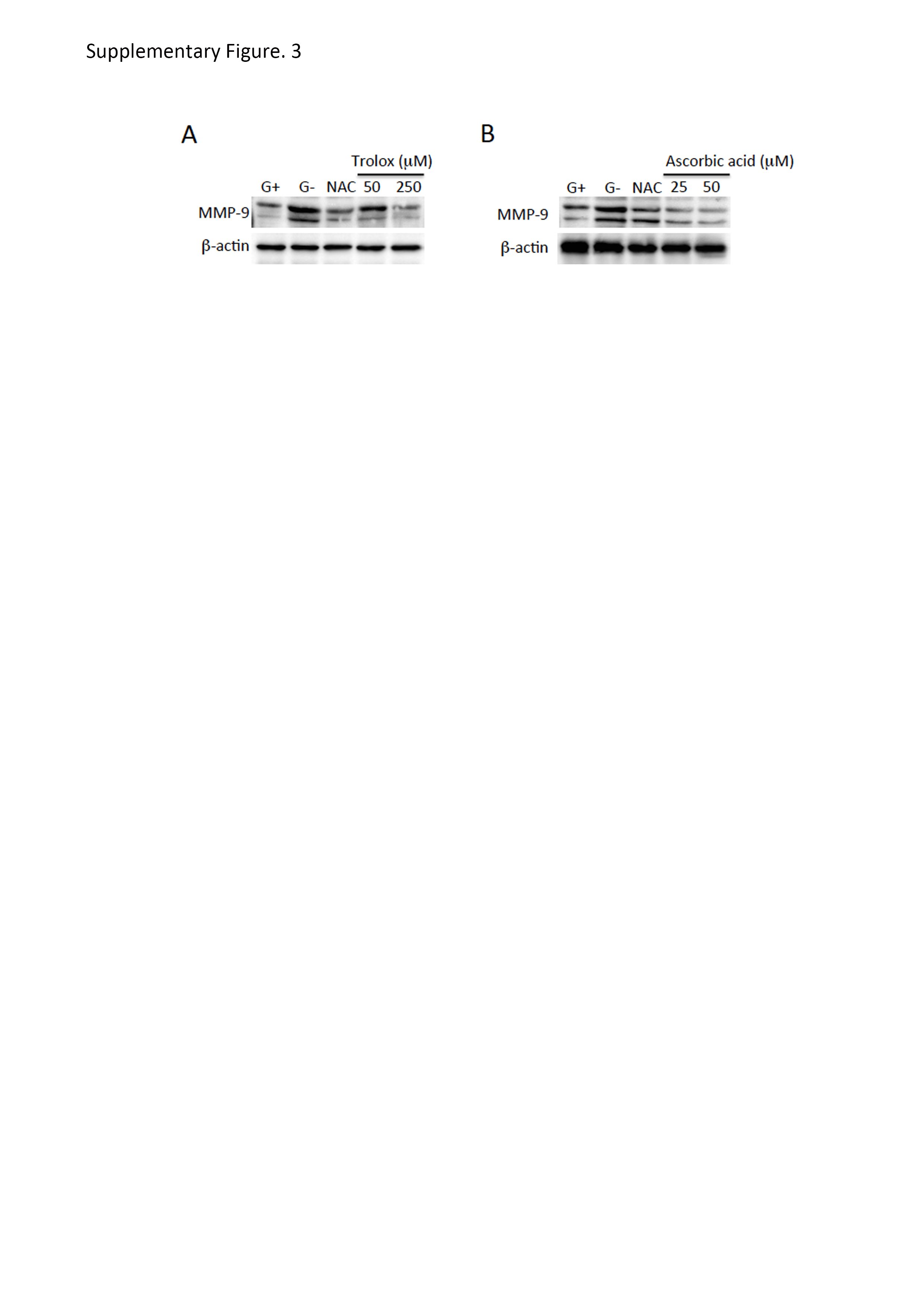


**Figure S3.** Glucose starvation-induced MMP-9 expression is also prevented by antioxidants other than NAC. HepG2 cells expressing sh-control, sh-LKB1, or sh-AMPK were incubated in glucose-free medium in the absence (G-) or presence (G+) of 5.5 mM glucose, or 2.5 mM NAC, or indicated doses of (A) Trolox or (B) ascorbic acid for 6 h. Western blot analysis was performed on protein extracts of these cells with antibodies against the indicated proteins with β-actin as a loading control. All data shown here are representative of 3 independent sets of experiments. Uncropped blots of these experiments are presented in supplementary figure 9.

**
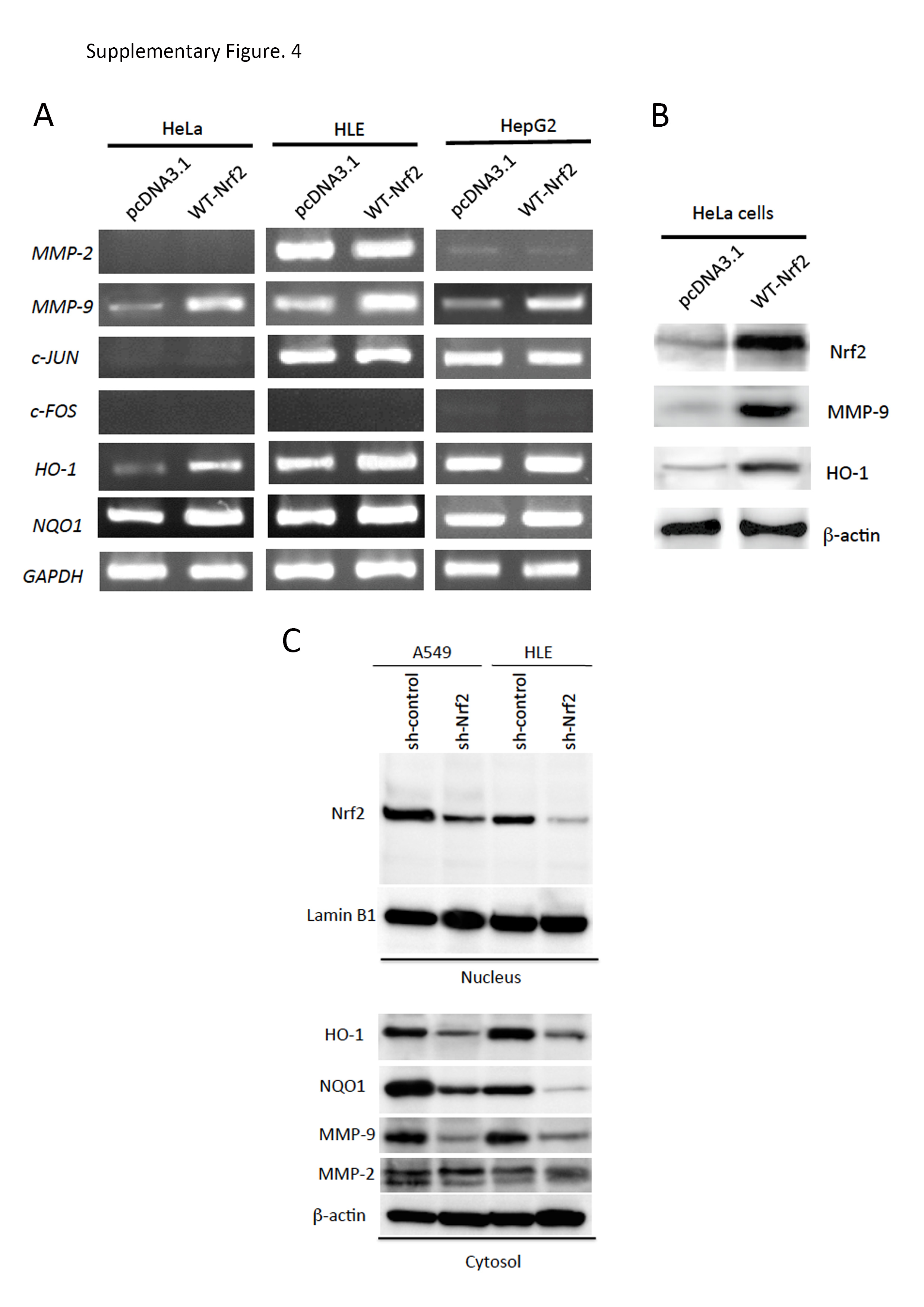
**

**Figure S4.** Nrf2 directly induces MMP-9 expression, but does not affect MMP-2 expression. (A) HeLa, HLE, and HepG2 cells were transiently transfected with empty pcDNA3.1 vector or WT-Nrf2 expression vector, and the expression levels of MMP-2, MMP-9, c-JUN, c-FOS, HO-1, NQO1, and GAPDH genes were examined by semiquantitative RT-PCR. (B) Western blots using antibodies against indicated proteins of whole-cell lysates of HeLa cells transiently transfected with pcDNA3.1 or WT-Nrf2 expression vector. (C) Whole-cell and nuclear lysates were examined by western blotting with antibodies against the indicated proteins in A549 and HLE cells expressing sh-control or sh-Nrf2. All data shown here are representative of 3 independent sets of experiments. Uncropped blots of these experiments are presented in supplementary figure 9.

**
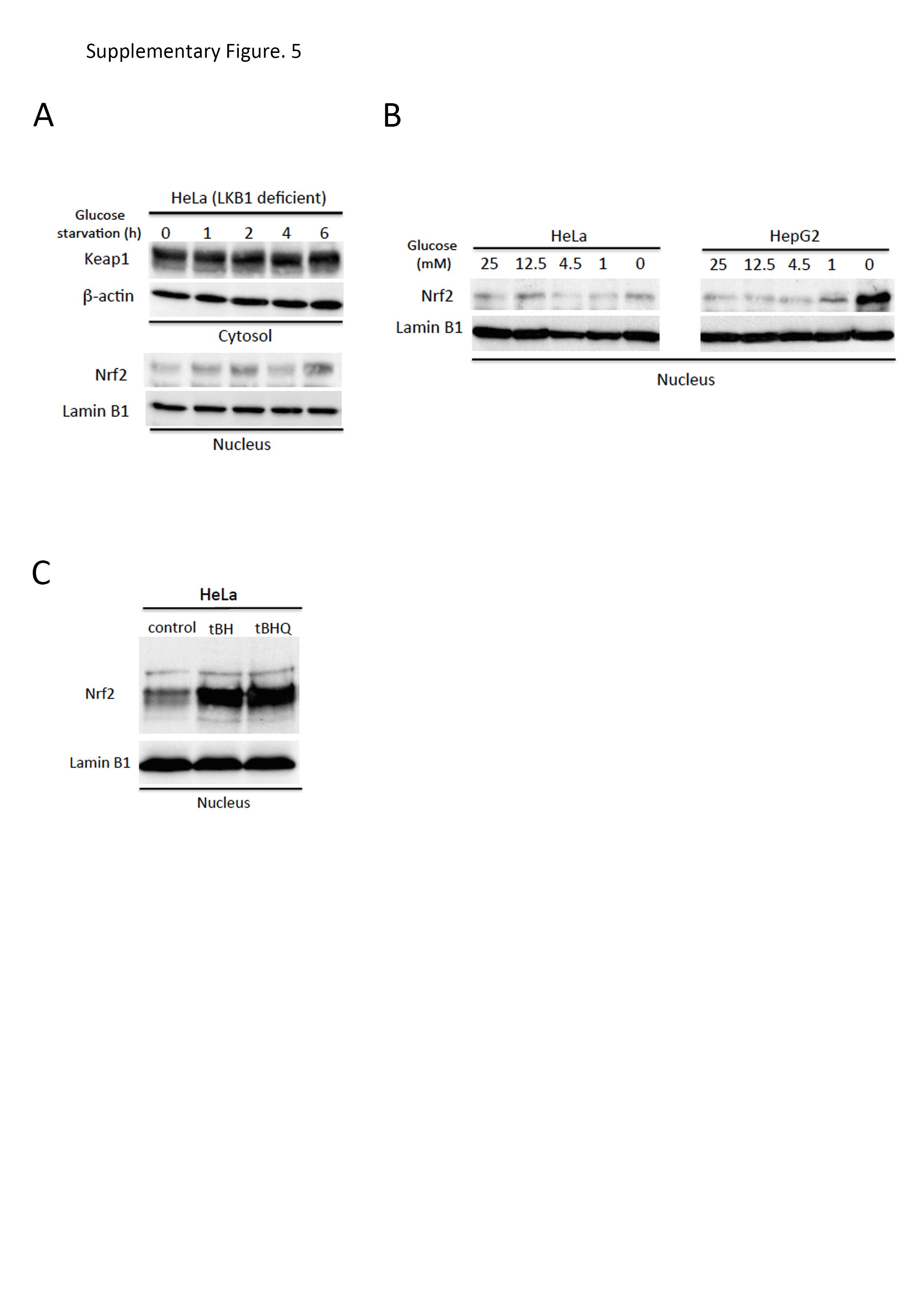
**

**Figure S5.** Glucose starvation fails to decrease Keap1 levels and Nrf2 induction in LKB1-deficient HeLa cells. Whole-cell or nuclear lysates of HeLa cells incubated in (A) absence of glucose for the indicated time periods and (B) at the indicated glucose concentrations were subjected to western blotting with antibodies against the indicated proteins. (C) HeLa cells were treated with 50 μM tBH or tBHQ for 6 h, following which the Nrf2 expression in nuclear lysates was analysed by western blotting. All data shown here are representative of 3 independent sets of experiments. Uncropped blots of all these experiments are presented in supplementary figure 9.


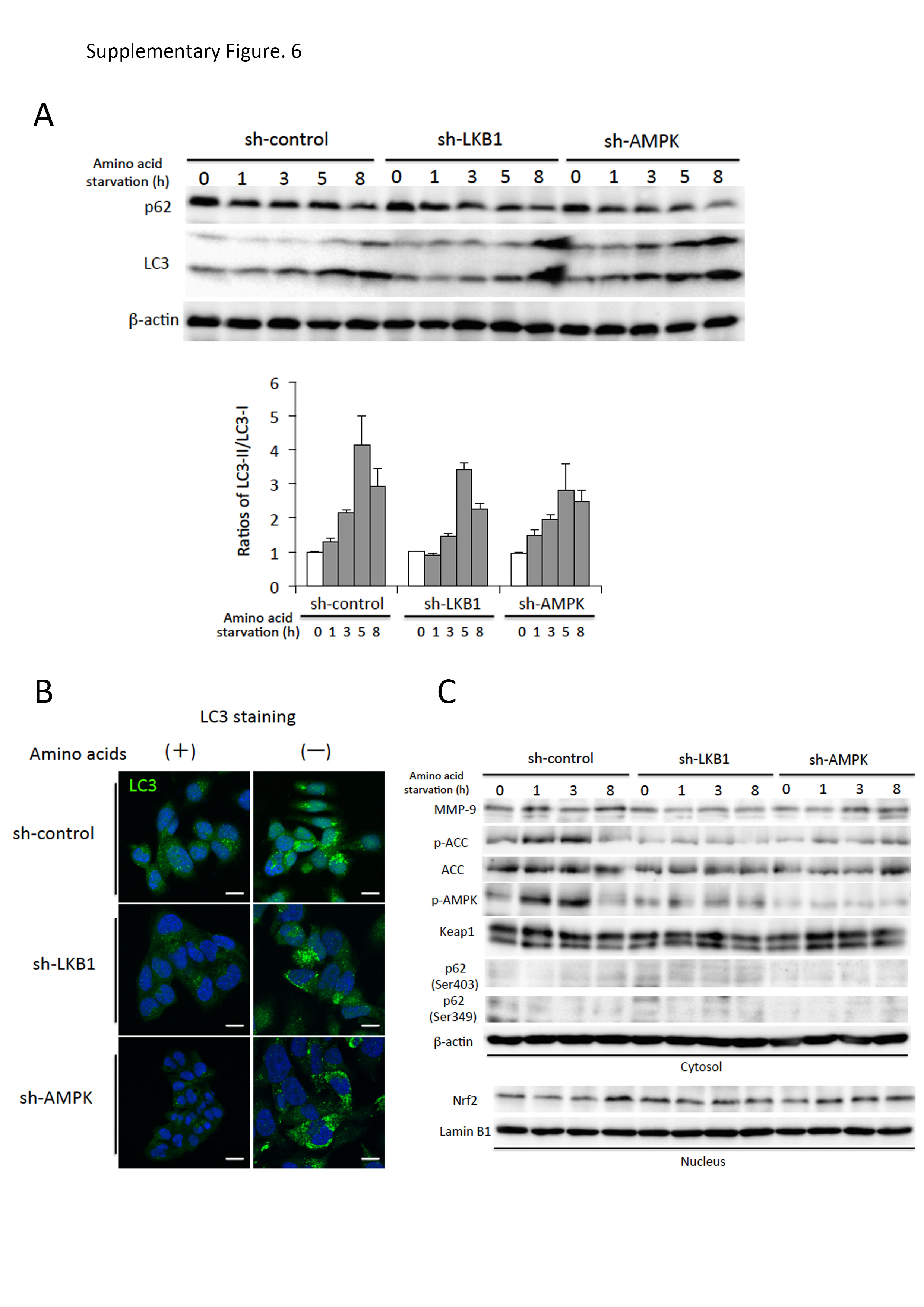


**Figure S6.** Amino acid starvation induces non-selective autophagy, but not MMP-9 expression, Nrf2 expression, or p62 phosphorylation in knockdowns of LKB1 or AMPK. (A) Whole-cell lysates of HepG2 cells expressing sh-control, sh-LKB1 or sh-AMPK and cultured in the amino acid-free medium, EBSS, for the indicated time periods were analysed by western blotting with antibodies against the indicated proteins with β-actin levels as loading controls. The ratios of LC3-II/LC-I were measured by densitometric analysis. (B) Immunofluorescence staining with anti-LC3 antibodies of HepG2 cells expressing sh-control, sh-LKB1, or sh-AMPK and incubated in amino acid-free EBSS in the absence (-) or presence (+) of MEM EAA and 2 mM L-glutamine for 6 h. The nucleus was stained with DAPI. The scale bar represents 10 μm. (C) Whole-cell and nuclear lysates of HepG2 cells expressing sh-control, sh-LKB1, or sh-AMPK and cultured in amino acid-free EBSS for the indicated time periods were analysed by western blotting with antibodies against the indicated proteins. All data shown here are representative of 3 independent sets of experiments. Uncropped blots of these experiments are presented in supplementary figure 9.


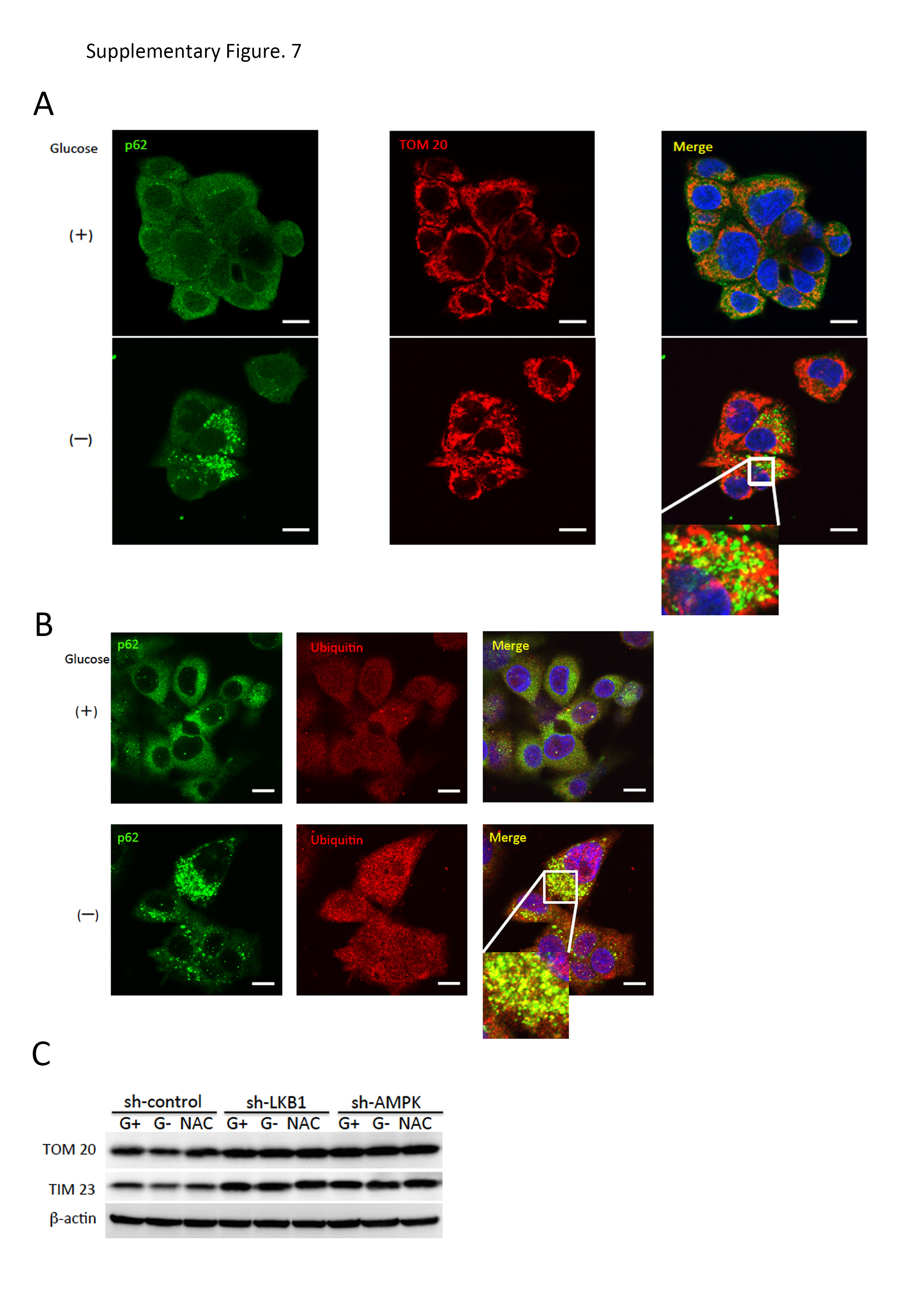


**Figure S7.** Under glucose starvation conditions, p62 was found to colocalise with mitochondria or ubiquitin. Immunofluorescence staining using antibodies against (A) p62 and TOM20, and (B) p62 and ubiquitin, of HepG2 cells incubated in glucose-free medium in the absence (-) or presence (+) of glucose for 6 h. The scale bar represents 10 μm. (C) Western blots of whole-cell lysates using antibodies against TOM 20 and TIM 23 of HepG2 cells expressing sh-control, sh-LKB1, or sh-AMPK and incubated in glucose-free medium in the absence (G-) or presence (G+) of 5.5 mM glucose, or 2.5 mM NAC for 6 h; β-actin levels were used as loading controls. All data shown here are representative of 3 independent sets of experiments. Uncropped blots of these experiments are presented in supplementary figure 9.


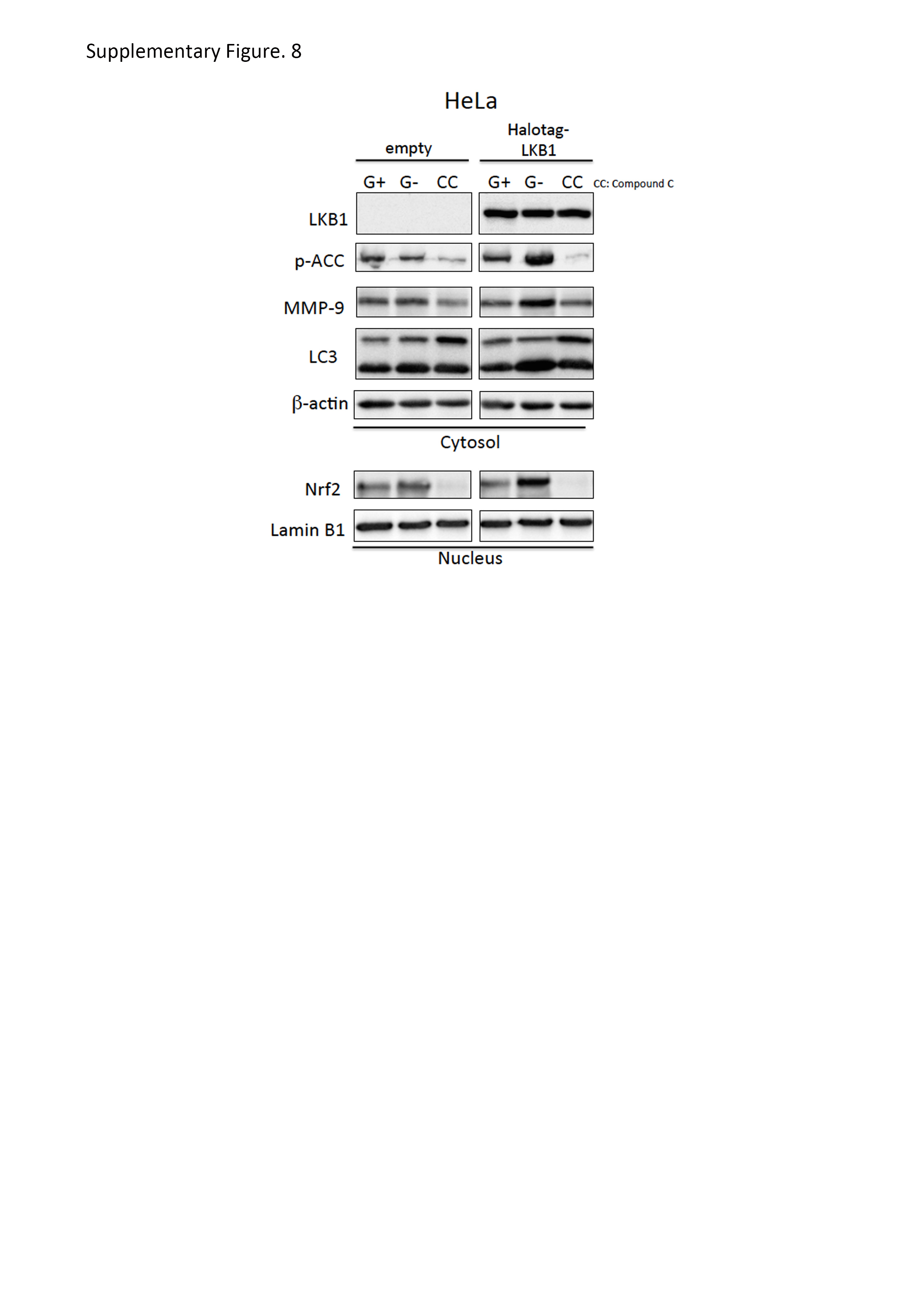


**Figure S8.** LKB1 reconstitution restores LKB1-AMPK-mediated LC3, MMP-9, and Nrf2 induction during glucose starvation in HeLa cells. HeLa cells stably expressing empty vectors or Halotag-fused LKB1 expression vectors were incubated in glucose-free medium in the absence (G-) or presence (G+) of 5.5 mM glucose, or 10 μM of compound C (CC) for 6 h. Whole-cell and nuclear lysates were analysed by western blotting with antibodies against the indicated proteins. All data shown here are representative of 3 independent sets of experiments. Uncropped blots of these experiments are presented in supplementary figure 9.

**
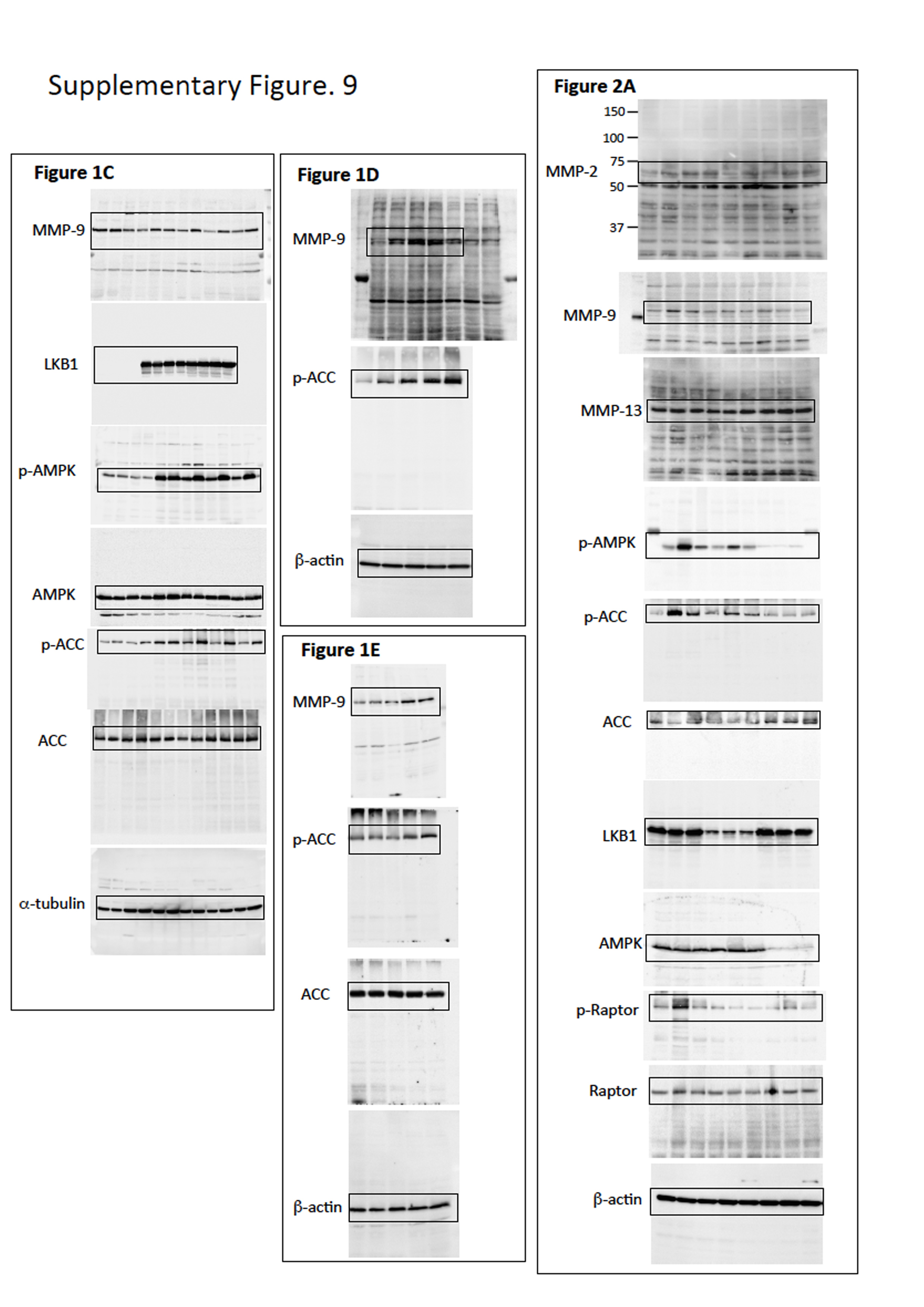
**

**
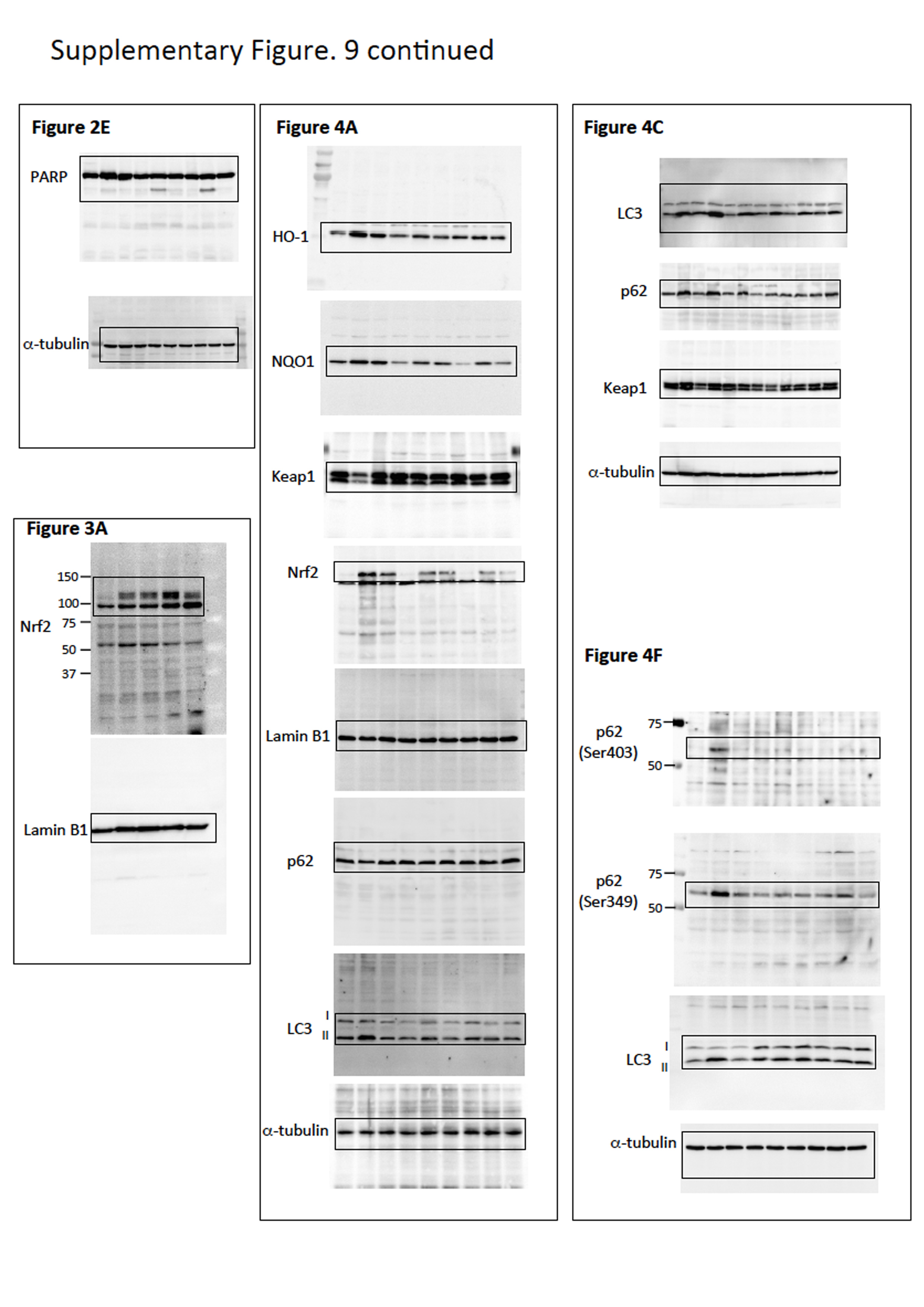
**

**
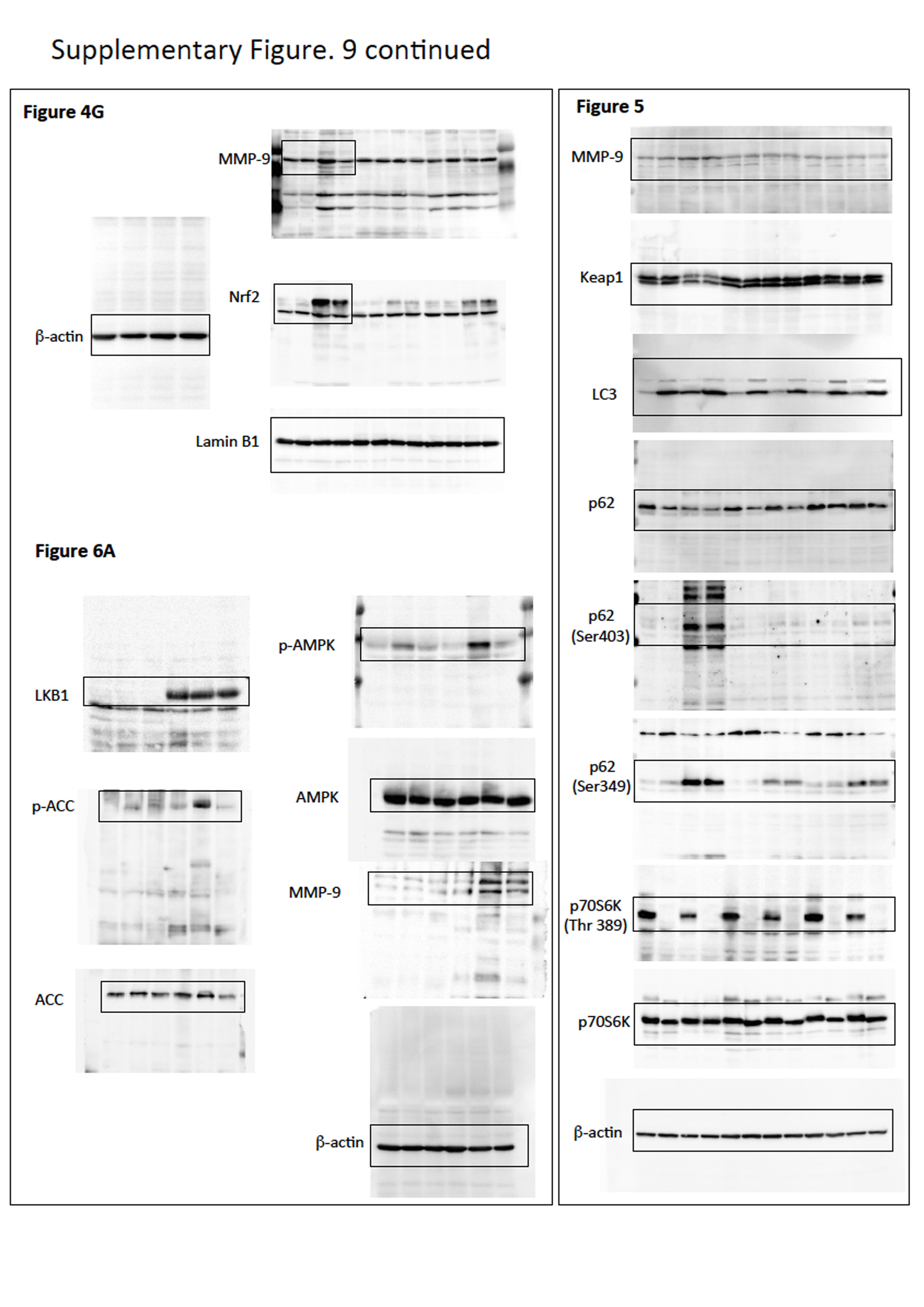
**

**
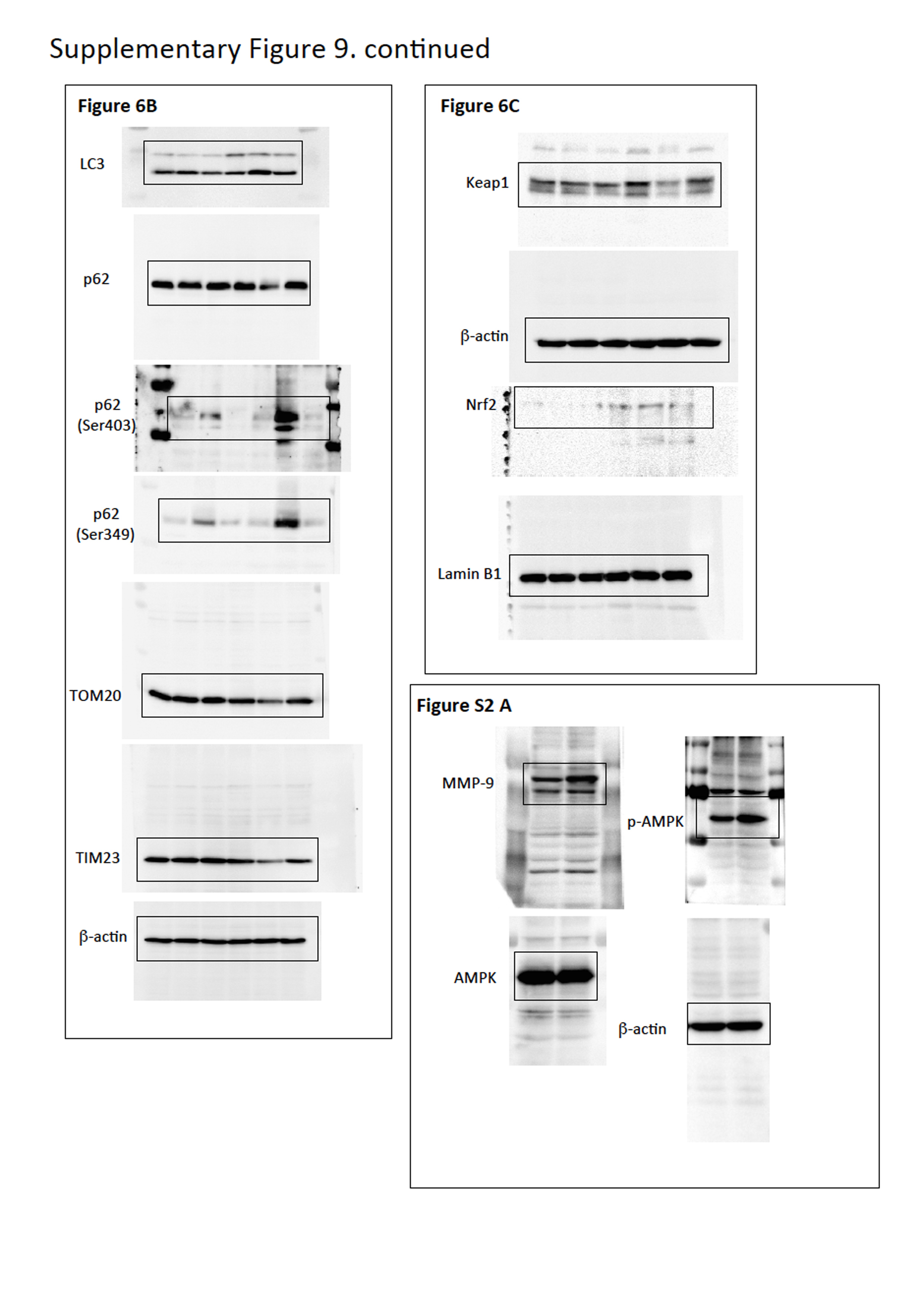
**

**
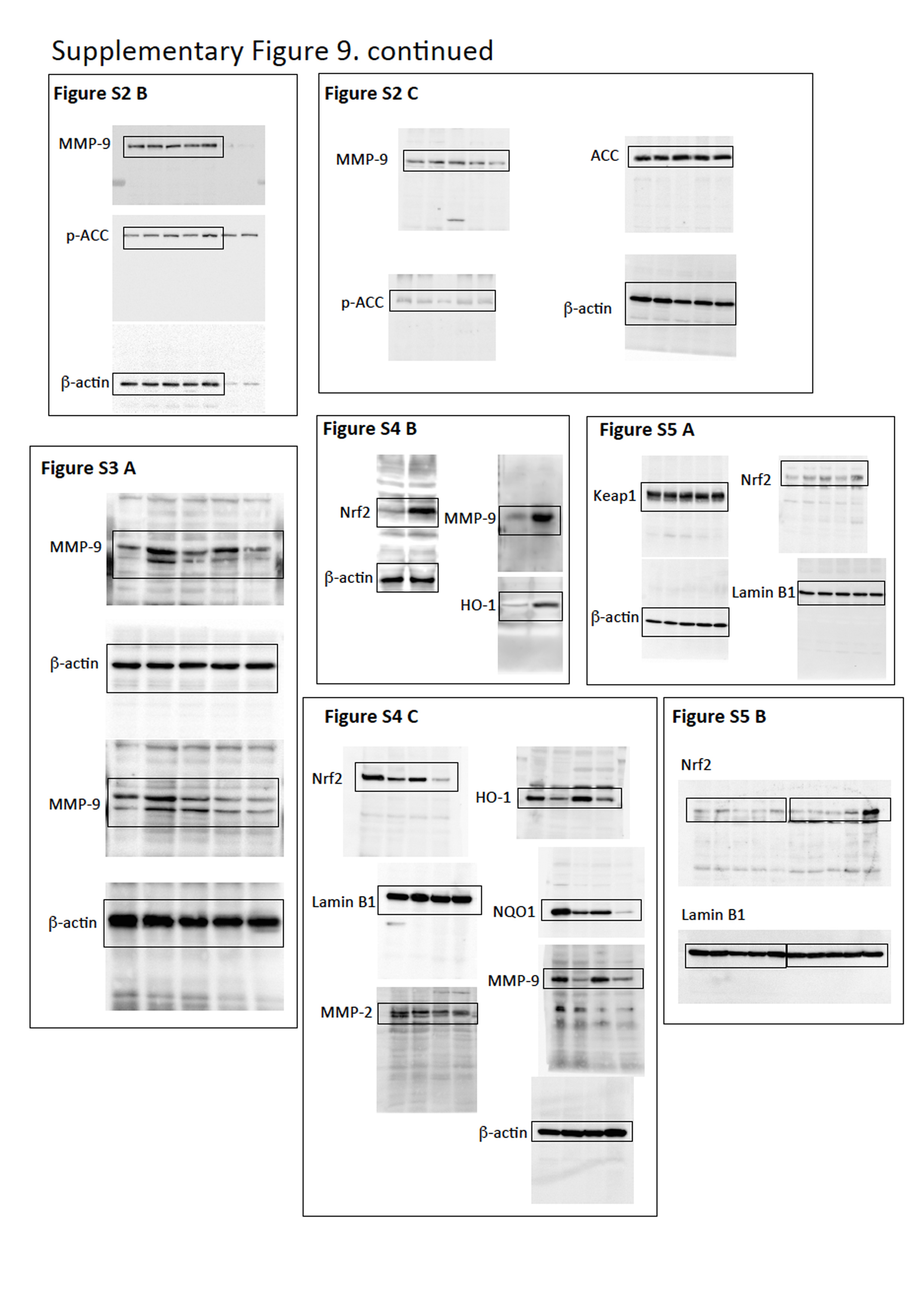
**

**
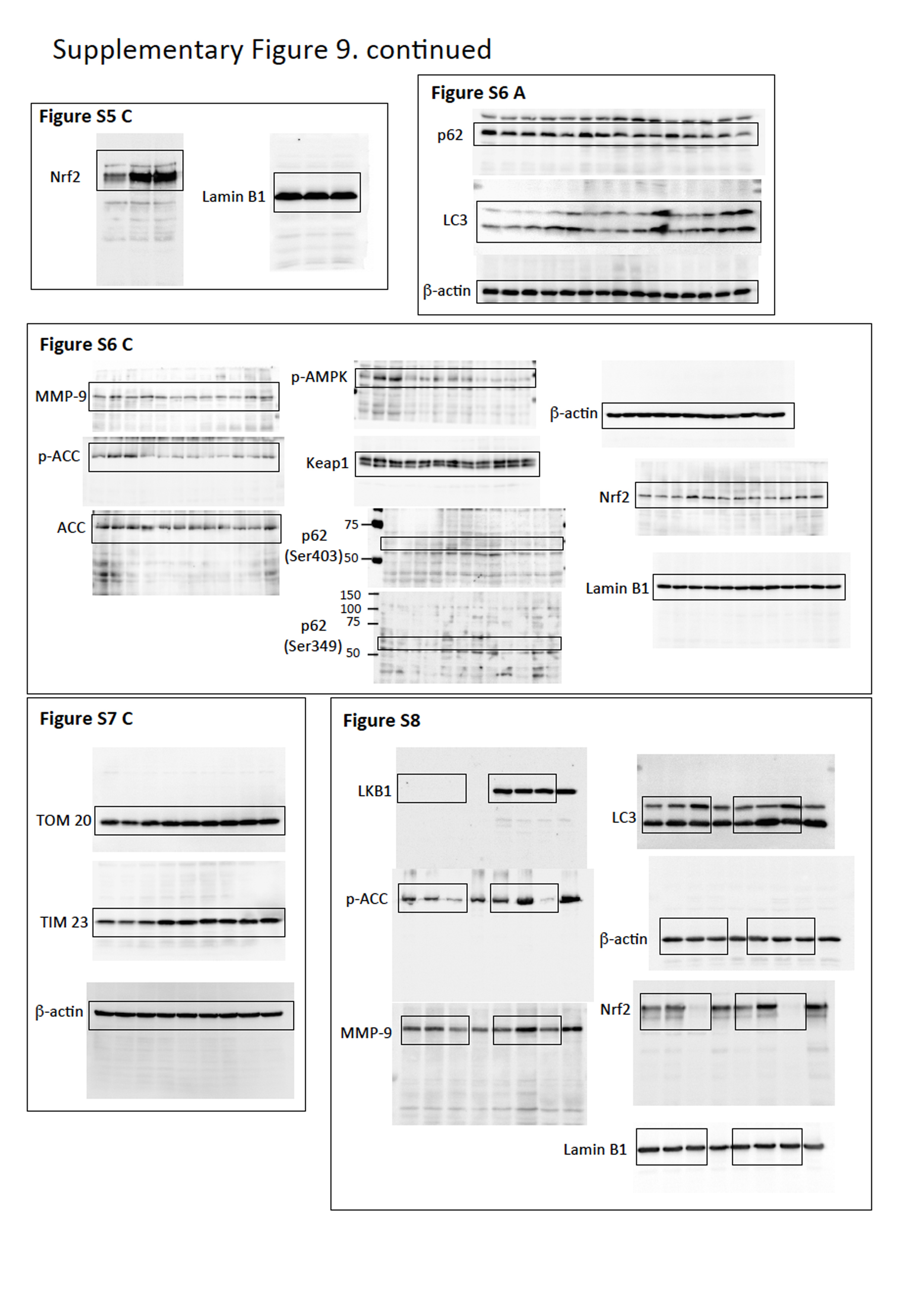
**

**Figure S9.** Uncropped western blots for all the immunoblot data presented in the main figures and supplementary figures. The full-length membranes were cut and incubated with the indicated antibodies to detect the proteins mentioned in the boxed regions of the figures.
